# SheddomeDB: the ectodomain shedding database for membrane-bound shed markers

**DOI:** 10.1186/s12859-017-1465-7

**Published:** 2017-03-14

**Authors:** Wei-Sheng Tien, Jun-Hong Chen, Kun-Pin Wu

**Affiliations:** 1 Institute of Biomedical Informatics, National Yang Ming University, Taipei, 112 Taiwan; 2 Bioinformatics Program, Taiwan International Graduate Program, Academia Sinica, Taipei, 115 Taiwan; 3 Department of Computer Science, National Taipei University of Education, Taipei, 106 Taiwan

**Keywords:** Ectodomain shedding, Biomarker discovery, Shed membrane proteins, Sheddome

## Abstract

**Background:**

A number of membrane-anchored proteins are known to be released from cell surface via ectodomain shedding. The cleavage and release of membrane proteins has been shown to modulate various cellular processes and disease pathologies. Numerous studies revealed that cell membrane molecules of diverse functional groups are subjected to proteolytic cleavage, and the released soluble form of proteins may modulate various signaling processes. Therefore, in addition to the secreted protein markers that undergo secretion through the secretory pathway, the shed membrane proteins may comprise an additional resource of noninvasive and accessible biomarkers. In this context, identifying the membrane-bound proteins that will be shed has become important in the discovery of clinically noninvasive biomarkers. Nevertheless, a data repository for biological and clinical researchers to review the shedding information, which is experimentally validated, for membrane-bound protein shed markers is still lacking.

**Results:**

In this study, the database SheddomeDB was developed to integrate publicly available data of the shed membrane proteins. A comprehensive literature survey was performed to collect the membrane proteins that were verified to be cleaved or released in the supernatant by immunological-based validation experiments. From 436 studies on shedding, 401 validated shed membrane proteins were included, among which 199 shed membrane proteins have not been annotated or validated yet by existing cleavage databases. SheddomeDB attempted to provide a comprehensive shedding report, including the regulation of shedding machinery and the related function or diseases involved in the shedding events. In addition, our published tool ShedP was embedded into SheddomeDB to support researchers for predicting the shedding event on unknown or unrecorded membrane proteins.

**Conclusions:**

To the best of our knowledge, SheddomeDB is the first database for the identification of experimentally validated shed membrane proteins and currently may provide the most number of membrane proteins for reviewing the shedding information. The database included membrane-bound shed markers associated with numerous cellular processes and diseases, and some of these markers are potential novel markers because they are not annotated or validated yet in other databases. SheddomeDB may provide a useful resource for discovering membrane-bound shed markers. The interactive web of SheddomeDB is publicly available at http://bal.ym.edu.tw/SheddomeDB/.

**Electronic supplementary material:**

The online version of this article (doi:10.1186/s12859-017-1465-7) contains supplementary material, which is available to authorized users.

## Background

A large class of proteins is known to be secreted from the cell to the extracellular space. The secreted proteins such as hormones, enzymes, and antibodies play vital regulatory roles in biological signaling and may serve as clinically noninvasive biomarkers and potential therapeutic targets [1, 2]. In addition to the proteins that undergo protein secretion via secretory pathways, membrane proteins are known to be released into the extracellular milieu via ectodomain shedding. Certain membrane-bound proteins, including cell adhesion molecules, growth factors, cytokines, and cell receptors, can be proteolytically cleaved by sheddase that results in the release of soluble forms of fragments. The process of ectodomain shedding has been shown to regulate various pathologies and diseases such as degeneration, inflammation, and cancer and physiological processes such as proliferation, differentiation, and migration [3, 4]. In this context, the cleaved and released membrane proteins resulting from shedding events may comprise additional resources of valuable secreted and soluble biomarkers for pathological states or physiological conditions.

Previous studies on membrane proteins revealed that only about 2 or 4% of cell surface molecules undergo the shedding process [5, 6]; hence, it is apparent that not every membrane protein will be released through proteolytic shedding. Therefore, to assess whether a membrane-bound protein will be released from cells and to identify membrane-bound shed markers that are of clinical potential, a data repository dedicated to provide shedding information that is experimentally validated for membrane proteins seems indispensable. Although cleavage databases such as MEROPS [7], PMAP-SubstrateDB [8], and HPRD [9] have been developed as information resources for proteases, substrates, and cleavage events, a portion of cleavage records collected by the databases may be based on library-based approaches for identification of protease cleavage sites [10, 11], and the putative substrates identified in the original literature may not be validated or physiologically relevant. In addition, currently, some shed membrane proteins that were identified by shedding studies may not have yet been recorded and annotated in these cleavage databases.

In this context, the database SheddomeDB was designed for the identification of shed membrane proteins that are released through proteolytic cleavage. The membrane proteins that were verified to be cleaved or released in the supernatant by immunological-based validation experiments were included in our database. Based on a comprehensive literature survey on shedding event studies, a total of 401 validated shed membrane proteins were identified, among which 199 shed membrane proteins have not been annotated or validated yet by current cleavage databases. SheddomeDB also provides a user-friendly web interface for researchers to search or browse proteins of interest. For each experimentally validated shed membrane protein, SheddomeDB attempted to provide a comprehensive shedding report based on literature references, including the regulation of shedding machinery and the related function or diseases involved in the shedding events. The cross-references to other resources, such as the released evidence in secretome data and the existing records of protease cleavage sites, were provided in SheddomeDB as well. In addition, the previously published prediction tool ShedP [12] was embedded into SheddomeDB. ShedP is a computational method developed to predict the shedding event on membrane proteins based on the protein sequence. By incorporating a prediction web interface for ShedP, SheddomeDB also supports the researchers for the assessment of shedding events on the unknown or unrecorded membrane proteins.

Thus, by collecting experimentally validated shed membrane proteins from literature references, SheddomeDB may provide a useful resource of membrane-bound shed markers associated with numerous cellular processes and diseases, including some potential novel markers that are not annotated or validated yet in other databases. SheddomeDB may be a useful bioinformatics design in sheddome marker discovery and to help investigate the regulatory role of membrane proteins in physiological and pathological processes. SheddomeDB is publicly available at http://bal.ym.edu.tw/SheddomeDB/.

## Methods

### Database implementation and interface design

The MYSQL relational database version 5.0.45 (http://www.mysql.com) was used in the current study to design and construct the SheddomeDB database and the interactive web interface. A JAVA-based model-view-controller (MVC) framework was utilized for the web interface to separate the logic, application, and the presentation into three distinct layers. All the interactions between the web client requests and the server side were handled by Apache web server. The dynamic web pages were designed using JavaServer Pages (JSP) and Cascading Style Sheets (CSS), and the user-interactive pages were supported by JavaScript and its library jQuery for client-side scripting.

### Data source

A comprehensive literature survey was conducted to identify the membrane proteins that were experimentally validated to be cleaved or released into the supernatant. By searching the PubMed database using the following keywords: “shedding”, “proteolytic”, “cleavage”, “protease”, “soluble form” and “released” the relevant studies on membrane protein shedding or protease cleavage were first acquired. We further manually reviewed the published studies and screened for the validated shed membrane proteins based on the following selection criteria: (1) The membrane proteins were verified to be cleaved by protease and protease inhibitors or the release of the soluble forms of proteins was detected in the culture supernatant. (2) The shedding events of membrane proteins were validated by antibody-based probes against the endogenous protein or against stably expressed genes encoding the protein. The curated publications that met the screening criteria were further selected as the data source to collect all the relevant data on membrane protein shedding. We mapped the membrane proteins in each curated publication to the UniProtKB/Swiss-Prot database [13] to uniform the protein ID based on the protein name and the organism source of the protein. In addition, because the functional consequences of membrane protein shedding can be diverse and depend on the protein function or the shed form of fragments, we grouped each shed membrane protein into functional categories based on the regulated functions or diseases suggested in the shedding literature. If the functional consequences of protein shedding were not clarified in the original studies, the shed membrane protein was then categorized based on the function description or annotation in the UniProtKB/Swiss-Prot database.

### Incorporated shedding predictor ShedP

The in-house prediction tool ShedP previously developed to predict shedding events of membrane proteins was incorporated into the SheddomeDB database. ShedP is a support vector machine (SVM)-based model [14] built by supervised machine learning that discriminates between shed membrane proteins and nonshed membrane proteins. The SVM model based on PseAAC [15] feature representation was constructed as our ShedP tool after a 5-fold cross-validation training procedure. At present, ShedP is the computational method published to predict shedding events of membrane proteins. To support the researchers for assessing the likelihood of an unknown or unrecorded membrane protein to be cleaved and released from the cell, we have also integrated a web interface to the prediction by ShedP into the SheddomeDB database, enabling valuable hints to be gained by *in silico* prediction.

## Results and Discussion

### Database content

In the present study, 436 curated studies were selected based on our literature survey process [3, 16–450], and a total of 401 validated shed membrane proteins were collected. Among the shed membrane proteins included in SheddomeDB, 22 proteins have not yet been annotated by existing cleavage databases MEROPS, PMAP-SubstrateDB, and HPRD. In addition, among those identified membrane proteins that have already been recorded in cleavage databases, 28 membrane proteins were only shown to undergo cleavage by signal peptidase [451–462] and 149 membrane proteins were only referenced by one substrate specificity study using a computational prediction model [463]. The cleavage records of membrane proteins in these studies may be neither relevant to membrane protein shedding nor experimentally validated. Therefore, our results revealed that a total of 199 shed membrane proteins in SheddomeDB were not annotated or validated yet by other cleavage databases. The details of the identified shed membrane proteins and the reference studies are summarized in (Additional file 1: Table S1).

Because the process of proteolytic shedding has been shown to be involved in various physiological processes and diseases, it is of importance to know which biological function categories or diseases may be regulated or related to the shedding of membrane proteins. Thus, we further grouped the validated shed membrane proteins into function categories manually based on the functional consequences referenced by shedding studies or functional description in the UniProtKB/Swiss-Prot database. First, the shed membrane proteins were grouped into the category “disease” if the proteins were shown to be involved in the disease progression or suggested as disease marker candidates. For instance, the shedding events of the proteins CDH1, EFNA1, and SDC1 were suggested to be involved in cancer invasion and immune escape [16–20]; the shedding events of SNCA and APP were shown to be involved in neurodegenerative disorders such as Parkinson’s disease and Alzheimer’s disease [21–23]; the shedding event of NRP2 was involved in immune disorders such as rheumatoid arthritis [24]; that of SDC4 was suggested to be involved in cardiovascular diseases such as atrial fibrillation [25]; HAVCR2 shedding event being implicated in HIV infection [26]; and the shedding event of CADM1 was shown to be involved in diabetes [27, 28]. In addition, several shed or soluble membrane proteins were suggested to be marker candidates for cancers (e.g., PVRL4 [29], CD200 [30, 31], CDH17 [32]), atherosclerosis (e.g., SORL1 [33–36]), diabetes mellitus (e.g., CLEC1B [37]), neurodegenerative disorders (e.g., PDGFRB [38, 39]), and hepatocyte damage (e.g., PTPRG [40]).

Then, a large portion of the shed membrane proteins were found to be related to immune response or neural signaling and were categorized into “immune and inflammation” or “central nervous system,” respectively. For instance, the shedding of numerous cytokines, cell receptors, and cell adhesion molecules was shown to be involved in leukocyte recruitment (e.g., CXCL16 [23, 41, 42], CDH5 [23, 41], TNFRSF8 [41, 43]), T-cell proliferation (e.g., Lag3 [41, 44]), and other immunological modulations (e.g., CR2 [45]). In contrast, the shedding of cell adhesion molecules, ligands, and cell receptors was involved in axon guidance (e.g., Epha4 [46], Neo1 [47]) and neurite outgrowth (e.g., NCAM1 [48–50]). In addition, another group of shed membrane proteins were found to be specifically related to the cardiovascular system and were further categorized into “angiogenesis” or “blood and homoeostasis.” For instance, the shedding of the proteins JAM3 and CLEC14A was suggested to be involved in the regulation of angiogenesis [51, 52], and the shedding of the proteins GP1BA and GP5 was suggested to be involved in the regulation of platelet hemostasis [23, 53].

**Fig. 1:**
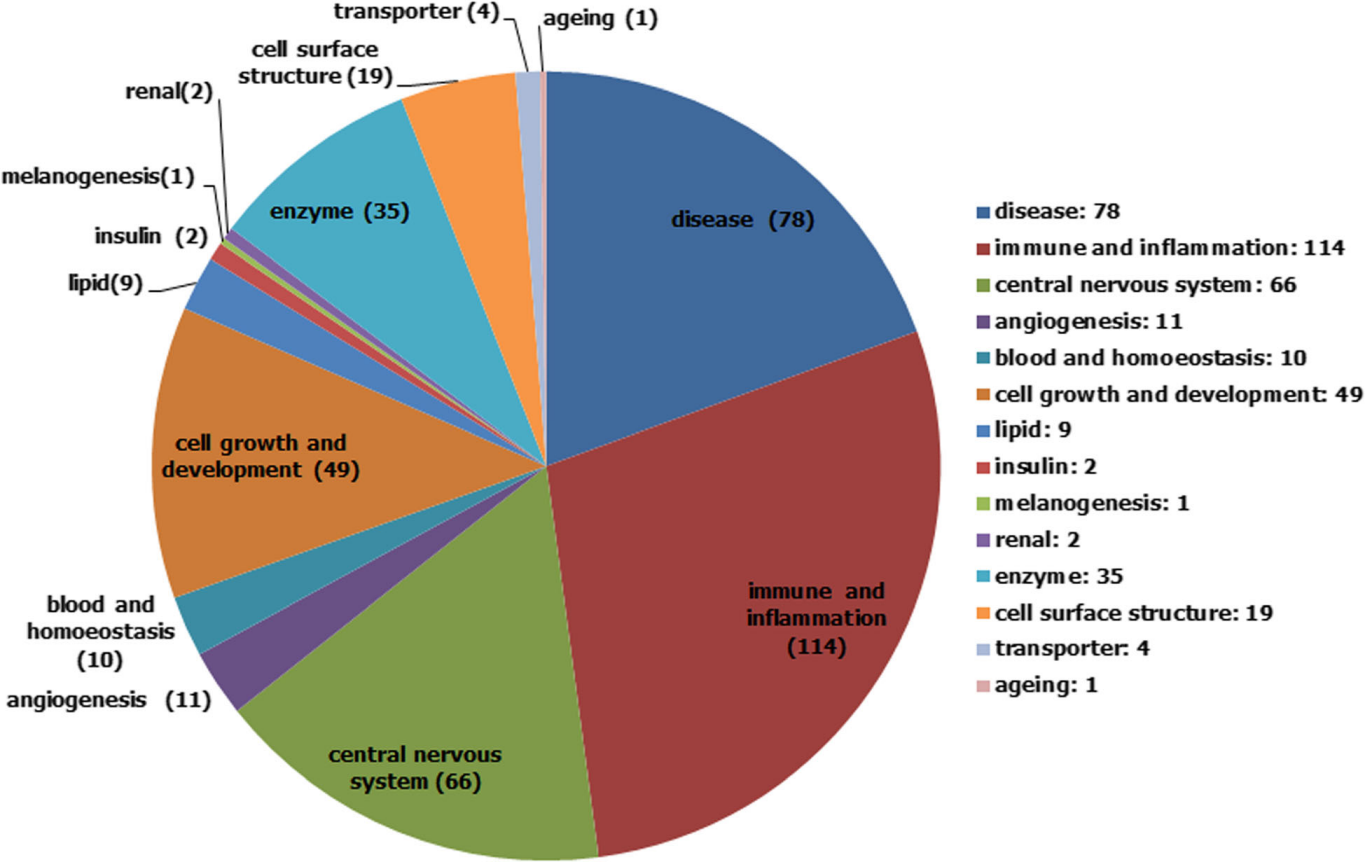
Function categorization of the identified 401 shed membrane proteins. The function category for membrane proteins was determined based on the functional consequences referenced by shedding studies or functional description in UniProtKB/Swiss-Prot. The protein numbers in each function category were revealed as well

For the remaining shed membrane proteins, most of the growth factors (e.g., BTC [23, 54, 55]), growth factor receptors (e.g., NGFR [56, 57]), morphogens (e.g., SHH [58]), and cell adhesion molecules (e.g., EPCAM [59, 60]) were shown to be involved in cell proliferation, migration, and morphogenesis and were categorized into “cell growth and development.” In addition, some shed proteins were found to be related to metabolism and were grouped into “lipid” (e.g., DLK1 [61, 62]), “melanogenesis” (e.g., PMEL [63]), “insulin” (e.g., TMEM27 [64, 65]), and “renal” (e.g., UMOD [66]). In addition, others were found to be related to protein function and were grouped into “enzyme” (e.g., ACE [67]), “transporter” (e.g., FOLR1 [68]), and “cell surface structure” (e.g., DSG2 [69]). Finally, we found some proteins that were specifically related to “aging” (e.g., KL [70, 71]). Thus, as depicted in Fig. 1, the 401 identified shed membrane proteins were grouped into 14 categories; the details of the protein members in each function category are summarized in (Additional file 1: Table S2) and can be reviewed in our browsed pages.

### User querying and web interface

**Fig. 2:**
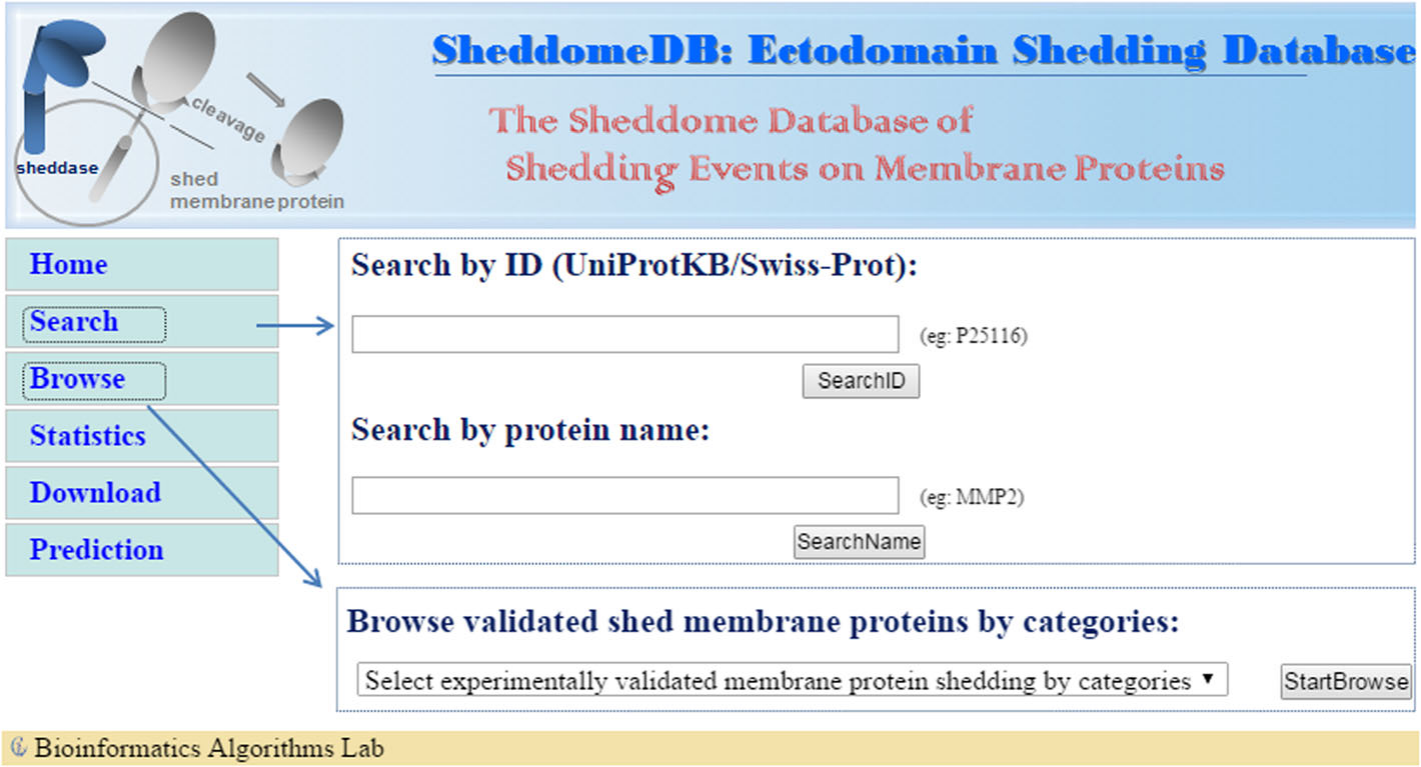
The interactive web interface of SheddomeDB. SheddomeDB provided a user-friendly web interface to query the database. The users can either search proteins of interest from the “Search” page or browse all shed membrane proteins from the “Browse” page

SheddomeDB provided a user-friendly web interface for researchers to search or browse proteins of interest. To query the database, the researchers can begin the search task from the “Search” page in which two query options were provided (Fig. 2). First, the database can be queried by directly specifying the protein UniProt ID. In contrast, the researchers can make a text similarity query by inputting the protein name or gene symbol and specify the desired one from all possible protein candidates in the interactive page. In addition, the researchers can choose the “Browse” page to browse the membrane proteins based on function categories (the disease or function categories involved in or related to shedding process) (Fig. 2).

**Fig. 3:**
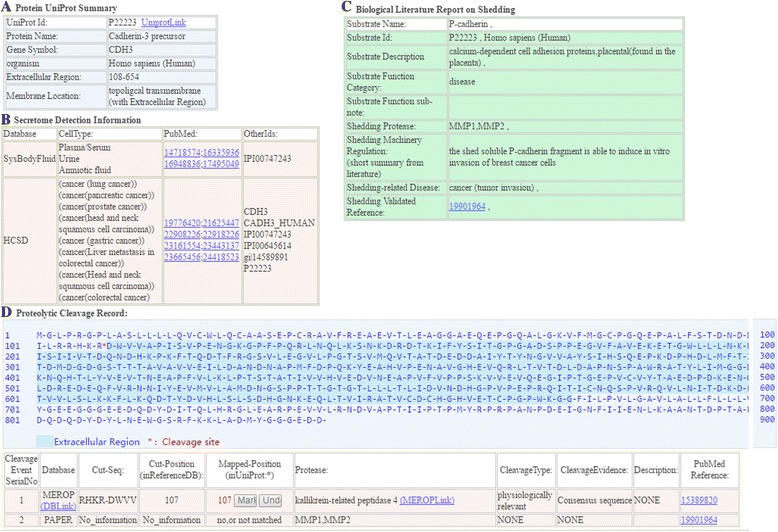
Example of SheddomeDB result pages. For each query membrane protein, the information was provided in four sections in the result pages. Section A revealed the basic protein information from UniProtKB/Swiss-Prot. Section B provided secretome released information. Section C summarized the biological information from the biologically validated literature on the shedding process. Section D provided existing cleavage site information. The protein sequence structure was depicted as well, in which the extracellular domain regions are marked with blue color and each protease cleavage site is labeled with an asterisk

In the results pages for each experimentally validated shed membrane protein, a comprehensive shedding report was provided by SheddomeDB in four sections (Fig. 3), as follows: (i) In section A, the basic information such as protein name, gene symbol, organism, and extracellular region were referenced from the UniProtKB/Swiss-Prot database. In addition, the membrane protein type annotated from UniProtKB/Swiss-Prot was also provided to show whether the membrane protein is (1.) lipid or GPI-anchored, (2.) topological (with extracellular and transmembrane domain, (3.) cell membrane annotated (annotated localized in cell membranes), and (4) other membrane proteins (proteins annotated at other subcellular localizations). (ii) The cross-references to the secretome released information were revealed in section B. If the query proteins were annotated to be released by secretome studies [72–74, 464–466] or by the secreted protein datasets in the databases the secretome databases HCSD [467] and Sys-BodyFluid [468], the secreted protein database SPD [469], and the subcellular localization database LOCATE [470], the secretome information such as the secretome database, secreted cell type, the reference PubMed ID, and the protein ID used in the reference literature will be summarized and provided. (iii) In section C, the regulation of the shedding machinery, the related function or disease, the protease name, and the PubMed ID of the shedding reference were summarized. (iv) In section D, the cross-references to existing cleavage sites records from current cleavage databases MEROPS, PMAP-SubstrateDB, and HPRD were provided. The cleavage information such as the cleavage database, protease name, reference PubMed ID, the cut location, and the cut sequence motif were provided. In addition, the protein sequence structure was represented in which the extracellular domain region and the protease cleavage site can be visualized.

### ShedP prediction interface

**Fig. 4:**
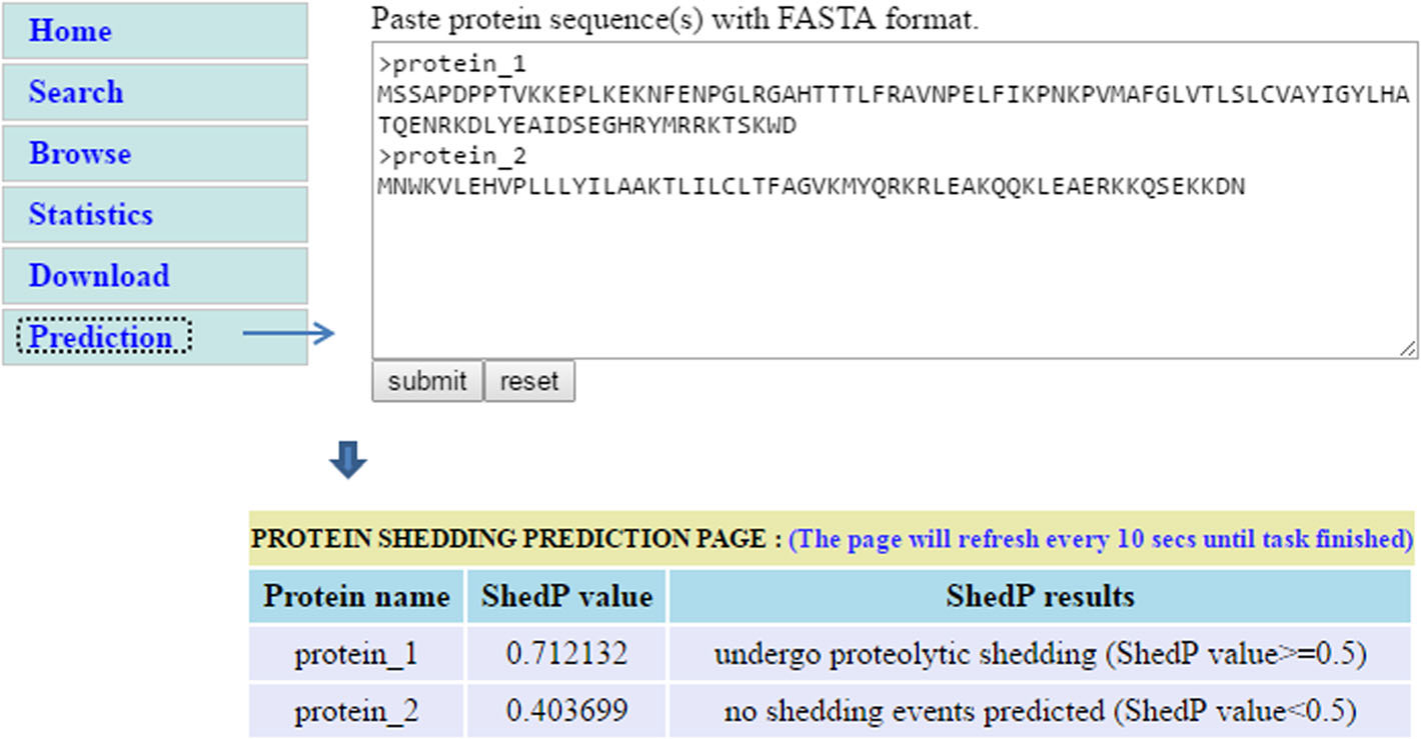
Web interface for ShedP prediction. To predict the shedding events, the users can insert the protein sequence (FASTA format) of a queried protein in “Prediction” pages. In the prediction result pages, the ShedP prediction results will be revealed. The protein will be regarded as positive and predicted to be shed if the ShedP prediction value is greater than or equal to 0.5, otherwise predicted as negative and nonshed

SheddomeDB incorporated a web interface to prediction by ShedP so as to gain valuable hints by *in silico* prediction. By inserting the protein sequence of a queried protein, the users can assess the likelihood of an unknown or unrecorded membrane protein to be cleaved and released from the cell. Based on the prediction model, a query protein whose predicted probability was greater than or equal to 0.5 was regarded as positive and predicted to be shed, otherwise it was predicted as negative and nonshed (Fig. 4).

## Conclusions

As more and more studies have revealed the regulatory role of ectodomain shedding in various cellular processes and pathologies, the identification of shed and released membrane proteins is becoming important in the field of biomarker discovery and sheddome proteomics. To determine and assess the possible membrane protein candidates undergoing shedding and released from the cells, the database SheddomeDB is the first sheddome-based database developed to store and query publicly available data on shed membrane proteins. For each queried membrane protein, SheddomeDB provides the researchers comprehensive cross-references including the released evidence in the secretome, protease cleavage record, and biologically validated shedding report. Thus, the bioinformatics-based database SheddomeDB may serve as a useful resource for membrane-bound secreted markers.

## Additional file

Additional file 1: Table S1. The details of the identified 401 shed membrane proteins including the protein UniProt ID and the PubMed ID for literature references. **Table S2.** The details of the shed membrane protein members in each group of function category. (PDF 303 kb)
