# Supplementary material for: SheddomeDB: the ectodomain shedding database for membrane-bound shed markers
Source: BMC Bioinformatics. 2017 Mar 14;18(Suppl 3):42. doi: 10.1186/s12859-017-1465-7 (PMC5374707; doi:10.1186/s12859-017-1465-7)
Supplement: Additional file 1: Table S1. — The details of the identified 401 shed membrane proteins including the protein UniProt ID and the PubMed ID for literature references. Table S2. The details of the shed membrane protein members in each group of function category. (PDF 303 kb) [file 12859_2017_1465_MOESM1_ESM.pdf]

SUPPLEMENTARY TABLE LEGENDS

Table S1. The details of the identified 401 shed membrane proteins including the protein UniProt ID and the PubMed ID for literature references

Table S2. The details of the shed membrane protein members in each group of function category

Table S1. The details of the identified 401 shed membrane proteins including the protein UniProt ID and the PubMed ID for literature references.

Protein Name	Gene Symbol	UniProtID (human)	UniProtID (other species)	PubMedID	Reference	Annotated in Cleavage Database
Acid-sensing ion channel 1	ASIC1	P78348	--	20601429	75	Not annotated (no reference in existing databases)
Beta-1 adrenergic receptor	ADRB1	P08588	--	23066091	76	Not annotated (no reference in existing databases)
Beta-galactoside alpha-2,6-sialyltransferase 1	St6gal1	--	P13721	2505760	77	Not annotated (no reference in existing databases)
Claudin-2	CLDN2	P57739	--	16232214,24665393	78,60	Not annotated (no reference in existing databases)
Claudin-5	Cldn5	--	O54942	26242473	79	Not annotated (no reference in existing databases)
Collagen alpha-1(XXIII) chain	COL23A1	Q86Y22	Q8K4G2	17627939,12644459	80,81	Not annotated (no reference in existing databases)
Collectin-12	COLEC12	Q5KU26	--	26290605	37	Not annotated (no reference in existing databases)
C-type lectin domain family 1 member B	CLEC1B	Q9P126	--	26290605	37	Not annotated (no reference in existing databases)
Endothelin-converting enzyme 1	ECE1	P42892	--	17761169	82	Not annotated (no reference in existing databases)
Mucin-16	MUC16	Q8WXI7	--	25205731	83	Not annotated (no reference in existing databases)
Neurotrimin precursor	Ntm	--	Q62718	25538237	84	Not annotated (no reference in existing databases)
Ninjurin-1	Ninj1	--	O70131	23142597	85	Not annotated (no reference in existing databases)
Suppressor of tumorigenicity 14 protein homolog	St14	--	P56677	25245289,20652801	86,87	Not annotated (no reference in existing databases)
Receptor-binding cancer antigen expressed on SiSo cells	EBAG9	O00559	--	25177692	88	Not annotated (no reference in existing databases)
Transferrin receptor protein 2	TFR2	Q9UP52	--	25637053	89	Not annotated (no reference in existing databases)
Transmembrane protease serine 6	TMPRSS6	Q8IU80	--	24867957	90	Not annotated (no reference in existing databases)
Tumor necrosis factor ligand superfamily member 9	TNFSF9	P41273	--	11564827	91	Not annotated (no reference in existing databases)
Tumor necrosis factor receptor superfamily member 13B	TNFRSF13B	O14836	--	25505277	92	Not annotated (no reference in existing databases)
Tumor necrosis factor receptor superfamily member 27	EDA2R	Q9HAV5	--	20501644	93	Not annotated (no reference in existing databases)
Type-1A angiotensin II receptor	Agtr1	--	P25095	20080964	94	Not annotated (no reference in existing databases)
Uncharacterized protein	SEMA5B	--	F1NSD7	22817385	95	Not annotated (no reference in existing databases)
Vasopressin V2 receptor	AVPR2	--	P48044	7896781	96	Not annotated (no reference in existing databases)

Angiopoietin-1 receptor precursor	TEK	Q02763	--	17901375,	143	Not shedding related (existing reference for signal peptidase)
Carbonic anhydrase 9 precursor	CA9	Q16790	--	9439598	238	Not shedding related (existing reference for signal peptidase)
CD226 antigen precursor	CD226	Q15762	--	19490613	239	Not shedding related (existing reference for signal peptidase)
CD276 antigen precursor	CD276	Q5ZPR3	--	18194267	240	Not shedding related (existing reference for signal peptidase)
CD83 antigen precursor	CD83	Q01151	--	18513799	241	Not shedding related (existing reference for signal peptidase)
Chondroitin sulfate proteoglycan 5 precursor	Cspg5	--	Q9ERQ6	17532789	242	Not shedding related (existing reference for signal peptidase)
C-type lectin domain family 14 member A precursor	CLEC14A	Q86T13	--	26939791	52	Not shedding related (existing reference for signal peptidase)
Endoglin precursor	ENG	P17813	--	20424116,22296769	243,244	Not shedding related (existing reference for signal peptidase)
Ephrin-A1 precursor	EFNA1	P20827		23686306	19	Not shedding related (existing reference for signal peptidase)
Fibroblast growth factor receptor-like 1 precursor	FGFRL1	Q8N441	--	19920134	245	Not shedding related (existing reference for signal peptidase)
HLA class I histocompatibility antigen, A-2 alpha chain precursor	HLA-A	P01892	--	17150042	246	Not shedding related (existing reference for signal peptidase)
Inactive tyrosine-protein kinase 7 precursor	PTK7	Q13308	--	23095747	247	Not shedding related (existing reference for signal peptidase)
Insulin-like growth factor 1 receptor precursor	IGF1R	P08069	--	17524361	248	Not shedding related (existing reference for signal peptidase)
Junctional adhesion molecule B precursor	JAM2	P57087	--	25367033	249	Not shedding related (existing reference for signal peptidase)
Junctional adhesion molecule C precursor	JAM3	Q9BX67	--	20592283,	51	Not shedding related (existing reference for signal peptidase)
Leucine-rich repeat transmembrane	FLRT2	O43155	--	21673655,	156	Not shedding related (existing reference for signal

protein FLRT2 precursor						peptidase)
Limbic system-associated membrane protein precursor	Lsamp	--	Q62813	25538237	84	Not shedding related (existing reference for signal peptidase)
Neuronal growth regulator 1 precursor	Negr1	--	Q9Z0J8	25538237	84	Not shedding related (existing reference for signal peptidase)
Platelet-derived growth factor receptor beta precursor	PDGFRB	P09619	P05622	20529858,26407747	38,39	Not shedding related (existing reference for signal peptidase)
Polymeric immunoglobulin receptor precursor	PIGR	P01833	--	3108385	250	Not shedding related (existing reference for signal peptidase)
Protein delta homolog 1 precursor	DLK1	P80370	Q09163	16809777,26312857	61,62	Not shedding related (existing reference for signal peptidase)
Receptor-type tyrosine-protein phosphatase zeta precursor	Ptprz1	--	B9EKR1	18713734	196	Not shedding related (existing reference for signal peptidase)
Reticulon-4 receptor precursor	RTN4R	Q9BZR6	Q99M75	15331667,16849393	49,50	Not shedding related (existing reference for signal peptidase)
Thrombomodulin precursor	THBD	P07204	--	20605193	251	Not shedding related (existing reference for signal peptidase)
Tyrosine-protein kinase receptor Tie-1 precursor	TIE1	P35590	--	17728252	252	Not shedding related (existing reference for signal peptidase)
Tyrosine-protein phosphatase non-receptor type substrate 1 precursor	Sirpa	--	P97797	24036914	253	Not shedding related (existing reference for signal peptidase)
Vascular cell adhesion protein 1 precursor	VCAM1	P19320	P29533	16565325,15949468	41,254	Not shedding related (existing reference for signal peptidase)
Vesicular integral-membrane protein VIP36 precursor	LMAN2	Q12907	--	22016386	255	Not shedding related (existing reference for signal peptidase)
Acetylcholinesterase precursor	ACHE	P22303	--	21214569	97	Not validated (existing reference based on prediction model)
Advanced glycosylation end product-specific receptor precursor	AGER	Q15109	--	18952609,	98	Not validated (existing reference based on prediction model)
Aminopeptidase N	ANPEP	P15144	--	12473585	99	Not validated (existing reference based on prediction

						model)
Atrial natriuretic peptide receptor 1 precursor	NPR1	P16066	--	2458716	100	Not validated (existing reference based on prediction model)
BDNF/NT-3 growth factors receptor precursor	Ntrk2	--	P15209	26712630	101	Not validated (existing reference based on prediction model)
Beta-1,4-galactosyltransferase 1	B4GALT1	P15291	--	3093147,17021253	102,103	Not validated (existing reference based on prediction model)
Brain-specific angiogenesis inhibitor 1 precursor	BAI1	O14514	--	22330140	104	Not validated (existing reference based on prediction model)
Cadherin-11 precursor	CDH11	P55287	--	26312857	62	Not validated (existing reference based on prediction model)
Cadherin-17 precursor	CDH17	Q12864	--	25336636	32	Not validated (existing reference based on prediction model)
Cadherin-related family member 1 precursor	Cdhr1	--	Q8VHP6	15284225,	105	Not validated (existing reference based on prediction model)
CAMPATH-1 antigen precursor	CD52	P31358	--	15329909	106	Not validated (existing reference based on prediction model)
Carcinoembryonic antigen-related cell adhesion molecule 1 precursor	CEACAM1	P13688	--	15259011,	107	Not validated (existing reference based on prediction model)
CD9 antigen	CD9	P21926	--	15576472,23447688	114,115	Not validated (existing reference based on prediction model)
CD160 antigen precursor	CD160	O95971	--	17237375	110	Not validated (existing reference based on prediction model)
CD48 antigen precursor	CD48	P09326	--	20833258,9418191,11513145	111,112,113	Not validated (existing reference based on prediction model)
CD97 antigen precursor	CD97	P48960	Q9Z0M6	15576472,23447688	114,115	Not validated (existing reference based on prediction model)
Cell surface glycoprotein MUC18 precursor	MCAM	P43121	--	19229070	116	Not validated (existing reference based on prediction model)
Ciliary neurotrophic factor receptor subunit alpha precursor	CNTFR	P26992	--	7681218	117	Not validated (existing reference based on prediction model)

Collectrin precursor	TMEM27	Q9HBJ8	--	22628310, 16330324	64,65	Not validated (existing reference based on prediction model)
Complement component C1q receptor precursor	CD93	Q9NPY3	--	16565325,16002728	41,118	Not validated (existing reference based on prediction model)
Complement receptor type 1 precursor	CR1	P17927	--	7957565	119	Not validated (existing reference based on prediction model)
Complement receptor type 2 precursor	CR2	P20023	--	12938215	45	Not validated (existing reference based on prediction model)
Contactin-2 precursor	Cntn2	--	Q61330	22728825	74	Not validated (existing reference based on prediction model)
C-X-C motif chemokine 16 precursor	CXCL16	Q9H2A7	Q8BSU2	16565325,23428418,18951988	41,42,23	Not validated (existing reference based on prediction model)
Dipeptidyl peptidase 4	DPP4	P27487	P28843	8878393,25217834	120,121	Not validated (existing reference based on prediction model)
Down syndrome cell adhesion molecule homolog precursor	Dscam	--	Q9ERC8	23300735	122	Not validated (existing reference based on prediction model)
Dyslexia-associated protein KIAA0319 precursor	KIAA0319	Q5VV43	--	20943657	123	Not validated (existing reference based on prediction model)
Ephrin type-A receptor 2 precursor	EPHA2	P29317	P52801	10958785	124	Not validated (existing reference based on prediction model)
Ephrin-A5 precursor	Efna5	--	O08543	18951988,16239146	23,125	Not validated (existing reference based on prediction model)
Epigen precursor	Epgn	--	Q924X1	18951988,17169360	23,126	Not validated (existing reference based on prediction model)
Fibroblast growth factor receptor 2 precursor	FGFR2	P21802	--	16230393	127	Not validated (existing reference based on prediction model)
Fibroblast growth factor receptor 3 precursor	Fgfr3	--	Q61851	21865593	128	Not validated (existing reference based on prediction model)
Fibrocystin precursor	PKHD1	P08F94	--	16956880,17470460	129,130	Not validated (existing reference based on prediction model)
Fms-related tyrosine kinase 3 ligand	Flt3lg	--	P49772	15077180	131	Not validated (existing reference based on prediction

precursor						model)
Golgi integral membrane protein 4	GOLIM4	O00461	--	20041192	132	Not validated (existing reference based on prediction model)
G-protein coupled receptor 124 precursor	GPR124	Q96PE1	--	16982628	133	Not validated (existing reference based on prediction model)
Granulocyte-macrophage colony-stimulating factor receptor subunit alpha precursor	CSF2RA	P15509	--	16565325	41	Not validated (existing reference based on prediction model)
Hemojuvelin precursor	HFE2	Q6ZVN8	--	20937842	134	Not validated (existing reference based on prediction model)
Hepatitis A virus cellular receptor 1 precursor	HAVCR1	Q96D42	Q5QNS5	22843853, 24286866	135,136	Not validated (existing reference based on prediction model)
Hepatitis A virus cellular receptor 2 precursor	HAVCR2	Q8TDQ0	--	25609823,	26	Not validated (existing reference based on prediction model)
High affinity nerve growth factor receptor precursor	NTRK1	P04629	--	12058067	137	Not validated (existing reference based on prediction model)
HLA class I histocompatibility antigen, alpha chain E precursor	HLA-E	P13747	--	16920947	138	Not validated (existing reference based on prediction model)
HLA class I histocompatibility antigen, alpha chain G precursor	HLA-G	P17693	--	14697234	139	Not validated (existing reference based on prediction model)
Immunoglobulin alpha Fc receptor precursor	FCAR	P24071	--	20059578	140	Not validated (existing reference based on prediction model)
Intercellular adhesion molecule 2 precursor	ICAM2	P13598	--	19524015	141	Not validated (existing reference based on prediction model)
Intercellular adhesion molecule 3 precursor	ICAM3	P32942	--	7876564	142	Not validated (existing reference based on prediction model)
Intercellular adhesion molecule 5 precursor	Icam5	--	Q60625	17901375	143	Not validated (existing reference based on prediction model)
Interleukin-1 receptor type 2 precursor	IL1R2	P27930	P27931	16565325,10210771	41,144	Not validated (existing reference based on prediction model)
Interleukin-11 receptor subunit	IL11RA	Q14626	--	26876177	145	Not validated (existing reference based on prediction

alpha precursor						model)
Interleukin-13 receptor subunit alpha-2 precursor	IL13RA2	Q14627	--	17603012,18694590	146,147	Not validated (existing reference based on prediction model)
Interleukin-2 receptor subunit beta	IL2RB	P14784	--	16565325,20495002	41,148	Not validated (existing reference based on prediction model)
Interleukin-23 receptor precursor	IL23R	Q5VWK5	--	26961870	149	Not validated (existing reference based on prediction model)
Interleukin-4 receptor subunit alpha precursor	IL4R	P24394	--	10341317	150	Not validated (existing reference based on prediction model)
Interleukin-5 receptor subunit alpha precursor	IL5RA	Q01344	--	12444155	151	Not validated (existing reference based on prediction model)
Interleukin-6 receptor subunit beta precursor	IL6ST	P40189	--	8353278	152	Not validated (existing reference based on prediction model)
Interleukin-7 receptor subunit alpha precursor	IL7R	P16871	--	17956896	153	Not validated (existing reference based on prediction model)
Kin of IRRE-like protein 2 precursor	Kirrel2	--	Q7TSU7	26324709	154	Not validated (existing reference based on prediction model)
Leptin receptor precursor	LEPR	P48357	--	11564702	155	Not validated (existing reference based on prediction model)
Leucine-rich repeat transmembrane protein FLRT1 precursor	FLRT1	Q9NZU1	--	21673655	156	Not validated (existing reference based on prediction model)
Leucine-rich repeat transmembrane protein FLRT3 precursor	FLRT3	Q9NZU0	--	21673655	156	Not validated (existing reference based on prediction model)
Leucine-rich repeat-containing protein 4B precursor	Lrrc4b	--	P0C192	24298159	157	Not validated (existing reference based on prediction model)
Leucine-rich repeats and immunoglobulin-like domains protein 1 precursor	LRIG1	Q96JA1	--	21087604,	158	Not validated (existing reference based on prediction model)
Leucine-rich repeats and immunoglobulin-like domains protein 2 precursor	LRIG2	O94898	--	20041192,25353163	132,159	Not validated (existing reference based on prediction model)

Leucine-rich repeats and immunoglobulin-like domains protein 3 precursor	LRIG3	Q6UXM1	--	20041192	132	Not validated (existing reference based on prediction model)
Low affinity immunoglobulin gamma Fc region receptor II-a precursor	FCGR2A	P12318	--	8254192	160	Not validated (existing reference based on prediction model)
Low affinity immunoglobulin gamma Fc region receptor III-A precursor	FCGR3A	P08637	--	23487023	161	Not validated (existing reference based on prediction model)
Low affinity immunoglobulin gamma Fc region receptor III-B precursor	FCGR3B	O75015	--	23228566	162	Not validated (existing reference based on prediction model)
Low-density lipoprotein receptor-related protein 4 precursor	Lrp4	--	Q8VI56	17227771	163	Not validated (existing reference based on prediction model)
Low-density lipoprotein receptor-related protein 8 precursor	LRP8	Q14114	--	25233900,15950758	164,165	Not validated (existing reference based on prediction model)
Lymphatic vessel endothelial hyaluronic acid receptor 1 precursor	LYVE1	Q9Y5Y7	--	26966180	166	Not validated (existing reference based on prediction model)
Lymphocyte function-associated antigen 3 precursor	CD58	P19256	--	9732704	167	Not validated (existing reference based on prediction model)
Macrophage colony-stimulating factor 1 receptor precursor	CSF1R	P07333	P09581	11160199	168	Not validated (existing reference based on prediction model)
Macrophage mannose receptor 1 precursor	MRC1	P22897	Q61830	10545481,9722572	169,170	Not validated (existing reference based on prediction model)
Mast/stem cell growth factor receptor Kit precursor	KIT	P10721	--	14625290,7536489	171,172	Not validated (existing reference based on prediction model)
Membrane primary amine oxidase	AOC3	Q16853	--	22618595	173	Not validated (existing reference based on prediction model)
MHC class I polypeptide-related sequence B precursor	MICB	Q29980	--	16698441	174	Not validated (existing reference based on prediction model)
Myeloid cell surface antigen CD33 precursor	CD33	P20138	--	24674885,20362641,16828866,	175,176,177	Not validated (existing reference based on prediction model)
Nectin-4 precursor	PVRL4	Q96NY8	--	15784625	29	Not validated (existing reference based on prediction model)

Nephrin precursor	NPHS1	O60500	--	18287402	178	Not validated (existing reference based on prediction model)
Neural cell adhesion molecule 1 precursor	NCAM1	P13591	--	15884014	48	Not validated (existing reference based on prediction model)
Neurofascin precursor	Nfasc	--	P97685	16360652	179	Not validated (existing reference based on prediction model)
Neuroligin-1 precursor	Nlgn1	--	Q99K10	23083742	180	Not validated (existing reference based on prediction model)
Neuronal cell adhesion molecule precursor	NRCAM	Q92823	--	16357171	181	Not validated (existing reference based on prediction model)
Neuropilin-1 precursor	Nrp1	--	P97333	18818406	182	Not validated (existing reference based on prediction model)
NKG2D ligand 2 precursor	ULBP2	Q9BZM5	--	16510567	183	Not validated (existing reference based on prediction model)
NT-3 growth factor receptor precursor	Ntrk3	--	Q6VNS1	12569366	184	Not validated (existing reference based on prediction model)
OX-2 membrane glycoprotein precursor	CD200	P41217	--	11309369,22875025	30,31	Not validated (existing reference based on prediction model)
PILR alpha-associated neural protein precursor	Pianp	--	Q6P1B3	27349870,26188512	185,186	Not validated (existing reference based on prediction model)
Platelet glycoprotein 4	Cd36	--	Q08857	8655633	187	Not validated (existing reference based on prediction model)
Plexin-B1 precursor	PLXNB1	O43157	--	12533544	188	Not validated (existing reference based on prediction model)
Plexin-B2 precursor	PLXNB2	O15031	--	12533544	188	Not validated (existing reference based on prediction model)
Pro-neuregulin-2, membrane-bound isoform precursor	NRG2	O14511	--	26027736	189	Not validated (existing reference based on prediction model)
Prosaposin receptor GPR37 precursor	GPR37	O15354	--	26869225	190	Not validated (existing reference based on prediction model)
Protein jagged-1 precursor	JAG1	P78504	--	22936788	16	Not validated (existing reference based on prediction

						model)
Protocadherin Fat 1 precursor	FAT1	Q14517	--	15922730,24625754	191,192	Not validated (existing reference based on prediction model)
Protocadherin gamma-B4 precursor	PCDHGB4	Q9UN71	--	19049889,16751190	193,194	Not validated (existing reference based on prediction model)
Protocadherin gamma-C3 precursor	PCDHGC3	Q9UN70	--	19049889,16751190	193,194	Not validated (existing reference based on prediction model)
Protocadherin-12 precursor	PCDH12	Q9NPG4	--	21402705	195	Not validated (existing reference based on prediction model)
P-selectin precursor	Selp	--	Q01102	16565325	41	Not validated (existing reference based on prediction model)
Receptor-type tyrosine-protein phosphatase alpha precursor	Ptpra	--	P18052	18713734	196	Not validated (existing reference based on prediction model)
Receptor-type tyrosine-protein phosphatase F precursor	PTPRF	P10586	--	16478662	197	Not validated (existing reference based on prediction model)
Receptor-type tyrosine-protein phosphatase gamma precursor	PTPRG	P23470	--	25775014	40	Not validated (existing reference based on prediction model)
Receptor-type tyrosine-protein phosphatase R precursor	Ptprr	--	Q62132	17147696	198	Not validated (existing reference based on prediction model)
Receptor-type tyrosine-protein phosphatase S precursor	Ptprs	--	Q64605	9245795	199	Not validated (existing reference based on prediction model)
Renin receptor precursor	Atp6ap2	--	Q9CYN9	19380613	200	Not validated (existing reference based on prediction model)
Roundabout homolog 1 precursor	ROBO1	Q9Y6N7	--	16740745	201	Not validated (existing reference based on prediction model)
Seizure 6-like protein 2 precursor	Sez6l2	--	Q4V9Z5	23430253	72	Not validated (existing reference based on prediction model)
Seizure 6-like protein precursor	Sez6l	--	Q6P1D5	23430253	72	Not validated (existing reference based on prediction model)
Seizure protein 6 precursor	Sez6	--	Q7TSK2	22728825	74	Not validated (existing reference based on prediction model)

Semaphorin-4D precursor	SEMA4D	Q92854	--	17244710	202	Not validated (existing reference based on prediction model)
Semaphorin-7A precursor	SEMA7A	O75326	--	20962327	73	Not validated (existing reference based on prediction model)
SLAM family member 5 precursor	Cd84	--	Q18PI6	23025437	203	Not validated (existing reference based on prediction model)
Sortilin precursor	SORT1	Q99523	--	16393139,21730062	426,204	Not validated (existing reference based on prediction model)
T-cell ecto-ADP-ribosyltransferase 1 precursor	Art2a	--	P17981	26209623	205	Not validated (existing reference based on prediction model)
T-cell immunoglobulin and mucin domain-containing protein 2 precursor	Timd2	--	Q8R183	24164679	206	Not validated (existing reference based on prediction model)
T-cell immunoglobulin and mucin domain-containing protein 4 precursor	Timd4	--	Q6U7R4	24286866	136	Not validated (existing reference based on prediction model)
T-cell surface antigen CD2 precursor	CD2	P06729	--	7722333	207	Not validated (existing reference based on prediction model)
T-cell surface glycoprotein CD5 precursor	CD5	P06127	--	10488739	208	Not validated (existing reference based on prediction model)
T-cell surface glycoprotein CD8 alpha chain precursor	CD8A	P01732	--	6203997,6193233,7722333	209,210,207	Not validated (existing reference based on prediction model)
T-cell-specific surface glycoprotein CD28 precursor	CD28	P10747	--	15086406	211	Not validated (existing reference based on prediction model)
TGF-beta receptor type-1 precursor	TGFBR1	P36897	--	19595713	212	Not validated (existing reference based on prediction model)
Thyrotropin receptor precursor	TSHR	P16473	--	18074395	213	Not validated (existing reference based on prediction model)
T-lymphocyte activation antigen CD86 precursor	CD86	P42081	--	11021528	214	Not validated (existing reference based on prediction model)
Toll-like receptor 2 precursor	TLR2	O60603	--	25531754	215	Not validated (existing reference based on prediction

						model)
Toll-like receptor 3 precursor	Tlr3	--	Q99MB1	25305318	216	Not validated (existing reference based on prediction model)
Toll-like receptor 4 precursor	Tlr4	--	Q9QUK6	16885150	217	Not validated (existing reference based on prediction model)
Toll-like receptor 9 precursor	TLR9	Q9NR96	Q9EQU3	18820679, 21604257	218,219	Not validated (existing reference based on prediction model)
Transferrin receptor protein 1	TFRC	P02786	--	2752136	220	Not validated (existing reference based on prediction model)
Transforming growth factor beta receptor type 3 precursor	TGFBR3	Q03167	--	24966170	221	Not validated (existing reference based on prediction model)
Trem-like transcript 1 protein precursor	TREML1	Q86YW5	--	16505478	222	Not validated (existing reference based on prediction model)
Triggering receptor expressed on myeloid cells 1 precursor	TREM1	Q9NP99	--	17785845	223	Not validated (existing reference based on prediction model)
Triggering receptor expressed on myeloid cells 2 precursor	TREM2	Q9NZC2	--	24078628	224	Not validated (existing reference based on prediction model)
Tumor necrosis factor receptor superfamily member 11A precursor	Tnfrsf11a	--	O35305	20118276	225	Not validated (existing reference based on prediction model)
Tumor necrosis factor receptor superfamily member 21 precursor	TNFRSF21	O75509	--	15118097	226	Not validated (existing reference based on prediction model)
Tumor necrosis factor receptor superfamily member 8 precursor	TNFRSF8	P28908	--	16565325,11120787	41,43	Not validated (existing reference based on prediction model)
Tumor-associated calcium signal transducer 2 precursor	Tacstd2	--	Q8BGV3	20551327	227	Not validated (existing reference based on prediction model)
Tyrosine-protein kinase Mer precursor	MERTK	Q12866	--	26316303	228	Not validated (existing reference based on prediction model)
Tyrosine-protein kinase receptor UFO precursor	AXL	P30530	Q00993	7822279,16227584	229,230	Not validated (existing reference based on prediction model)
Tyrosine-protein kinase RYK precursor	Ryk	--	Q01887	19000841	231	Not validated (existing reference based on prediction model)

Uromodulin precursor	UMOD	P07911	--	11741296	66	Not validated (existing reference based on prediction model)
Vascular endothelial growth factor receptor 1 precursor	FLT1	P17948	--	19276374,	232	Not validated (existing reference based on prediction model)
Vascular endothelial growth factor receptor 2 precursor	KDR	P35968	--	20814017,	233	Not validated (existing reference based on prediction model)
Vasorin precursor	VASN	Q6EMK4	--	21170088,	234	Not validated (existing reference based on prediction model)
VPS10 domain-containing receptor SorCS1 precursor	SORCS1	Q8WY21	--	16393139	426	Not validated (existing reference based on prediction model)
VPS10 domain-containing receptor SorCS2 precursor	SORCS2	Q96PQ0	--	16393139	426	Not validated (existing reference based on prediction model)
VPS10 domain-containing receptor SorCS3 precursor	SORCS3	Q9UPU3	--	16393139	426	Not validated (existing reference based on prediction model)
V-set domain-containing T-cell activation inhibitor 1 precursor	VTCN1	Q7Z7D3	--	24848066	235	Not validated (existing reference based on prediction model)
Zinc transporter ZIP10 precursor	Slc39a10	--	Q6P5F6	22687393	236	Not validated (existing reference based on prediction model)
Zinc transporter ZIP4 precursor	Slc39a4	--	Q78IQ7	18936158	237	Not validated (existing reference based on prediction model)
40S ribosomal protein SA	RPSA	P08865	--	8655633	187	Annotated (existing reference for shedding information)
5'-nucleotidase	NT5E	P21589	--	9015312	256	Annotated (existing reference for shedding information)
72 kDa type IV collagenase precursor	MMP2	P08253	--	8663332	257	Annotated (existing reference for shedding information)
Adhesion G protein-coupled receptor E2 precursor	ADGRE2	Q9UHX3	--	16982628,12860403	133,258	Annotated (existing reference for shedding information)
Alpha-synuclein	SNCA	P37840	--	15863497,	21	Annotated (existing reference for shedding information)
Amiloride-sensitive sodium channel subunit alpha	Scnn1a	--	Q61180	15007080	259	Annotated (existing reference for shedding information)
Amiloride-sensitive sodium channel subunit gamma	Scnn1g	--	Q9WU39	15007080,18650438	259,260	Annotated (existing reference for shedding information)
Amphiregulin precursor	AREG	P15514	P31955	16769815,15337756, 24520077	261,262,263	Annotated (existing reference for shedding information)

Amyloid beta A4 protein precursor	APP	P05067	P12023	22553493,18951988	22,23	Annotated (existing reference for shedding information)
Amyloid-like protein 1 precursor	APLP1	P51693	--	9428684,12228233	264,265	Annotated (existing reference for shedding information)
Amyloid-like protein 2 precursor	APLP2	Q06481	--	16279945,12228233	266,265	Annotated (existing reference for shedding information)
Angiotensin-converting enzyme 2 precursor	ACE2	Q9BYF1	--	15983030,18490652, 17150042	267,268,246	Annotated (existing reference for shedding information)
Angiotensin-converting enzyme precursor	ACE	P12821	--	10187843	67	Annotated (existing reference for shedding information)
Annexin A1	ANXA1	P04083	--	17023068	269	Annotated (existing reference for shedding information)
Annexin A2	ANXA2	P07355	--	18721140	270	Annotated (existing reference for shedding information)
Atrial natriuretic peptide-converting enzyme	CORIN	Q9Y5Q5	--	21288900	271	Annotated (existing reference for shedding information)
Basal cell adhesion molecule precursor	BCAM	P50895	--	25051049	272	Annotated (existing reference for shedding information)
Basigin precursor	BSG	P35613	P26453	14985463,17050542,1714914	273,274,275	Annotated (existing reference for shedding information)
Beta-secretase 1 precursor	BACE1	P56817	P56818	12857759	276	Annotated (existing reference for shedding information)
Beta-secretase 2 precursor	BACE2	Q9Y5Z0	--	11316808	277	Annotated (existing reference for shedding information)
Cadherin-1 precursor	CDH1	P12830	Q9R0T4	22936788, 25967040, 24712279, 19049889	16,17,18,193	Annotated (existing reference for shedding information)
Cadherin-2 precursor	CDH2	P19022	P15116	19049889,15692570,18951988,16998833	193,278,23,279	Annotated (existing reference for shedding information)
Cadherin-3 precursor	CDH3	P22223	--	19901964,	280	Annotated (existing reference for shedding information)
Cadherin-5 precursor	CDH5	P33151	--	16565325,18951988	41,23	Annotated (existing reference for shedding information)
Calcium-activated chloride channel regulator 4A precursor	Clca4a	--	Q6Q473	22350745	281	Annotated (existing reference for shedding information)
Calsyntenin-1 precursor	CLSTN1	O94985	Q9DDD3	19864413,11161476	282,283	Annotated (existing reference for shedding information)
Calsyntenin-2 precursor	CLSTN2	Q9H4D0	--	19864413,	282	Annotated (existing reference for shedding information)
Calsyntenin-3 precursor	CLSTN3	Q9BQT9	--	19864413,	282	Annotated (existing reference for shedding information)
Cation-independent mannose-6-phosphate receptor precursor	IGF2R	P11717	--	2458716	100	Annotated (existing reference for shedding information)
CD109 antigen precursor	CD109	Q6YHK3	--	20035377	284	Annotated (existing reference for shedding information)
CD166 antigen precursor	ALCAM	Q13740	--	18171982	285	Annotated (existing reference for shedding information)
CD27 antigen precursor	CD27	P26842	--	1311261	286	Annotated (existing reference for shedding information)

CD40 ligand	CD40LG	P29965	--	18951988	23	Annotated (existing reference for shedding information)
CD44 antigen precursor	CD44	P16070	P15379	16565325,15197174,18951988	41,287,23	Annotated (existing reference for shedding information)
CD59 glycoprotein precursor	CD59	P13987	--	7558140	288	Annotated (existing reference for shedding information)
Cell adhesion molecule 1 precursor	CADM1	Q9BY67	Q8R5M8	26231557, 24964098, 22172944	27,28,289	Annotated (existing reference for shedding information)
Chondroitin sulfate proteoglycan 4 precursor	CSPG4	Q6UVK1	Q00657	15866049	290	Annotated (existing reference for shedding information)
Collagen alpha-1(XIII) chain	COL13A1	Q5TAT6	--	15005656	291	Annotated (existing reference for shedding information)
Collagen alpha-1(XVII) chain	COL17A1	Q9UMD9	--	17545155	292	Annotated (existing reference for shedding information)
Collagen alpha-1(XXV) chain	COL25A1	Q9BXS0	--	11927537,	293	Annotated (existing reference for shedding information)
Complement component 1 Q subcomponent-binding protein, mitochondrial precursor	C1QBP	Q07021	--	11773076	294	Annotated (existing reference for shedding information)
Complement decay-accelerating factor precursor	CD55	P08174	--	2477368	295	Annotated (existing reference for shedding information)
Coxsackievirus and adenovirus receptor homolog precursor	Cxadr	--	P97792	24015300	296	Annotated (existing reference for shedding information)
C-type lectin domain family 3 member A precursor	CLEC3A	O75596	--	19173304	297	Annotated (existing reference for shedding information)
CUB domain-containing protein 1 precursor	CDCP1	Q9H5V8	--	20551327	227	Annotated (existing reference for shedding information)
Cysteine-rich motor neuron 1 protein precursor	CRIM1	Q9NZV1	--	12805376,25453556	298,299	Annotated (existing reference for shedding information)
Cytokine receptor common subunit gamma precursor	Il2rg	--	P34902	11133759	300	Annotated (existing reference for shedding information)
Delta-like protein 1 precursor	Dll1	--	Q61483	18342566,18951988	301,23	Annotated (existing reference for shedding information)
Desmocollin-3 precursor	DSC3	Q14574	--	11500511,24665393	302,60	Annotated (existing reference for shedding information)
Desmoglein-1 precursor	DSG1	Q02413	--	26224314,24665393	69,60	Annotated (existing reference for shedding information)
Desmoglein-2 precursor	DSG2	Q14126	--	26224314	69	Annotated (existing reference for shedding information)
Desmoglein-3 precursor	DSG3	P32926	--	11500511,24665393	302,60	Annotated (existing reference for shedding information)
Dipeptidase 1 precursor	DPEP1	P16444	--	11988094	303	Annotated (existing reference for shedding information)
Disintegrin and metalloproteinase	ADAM10	O14672	O35598	26554003	307	Annotated (existing reference for shedding information)

domain-containing protein 10 precursor						
Disintegrin and metalloproteinase domain-containing protein 15 precursor	ADAM15	Q13444	--	25208722	304	Annotated (existing reference for shedding information)
Disintegrin and metalloproteinase domain-containing protein 19 precursor	ADAM19	Q9H013	--	12393862	305	Annotated (existing reference for shedding information)
Disintegrin and metalloproteinase domain-containing protein 28 precursor	ADAM28	Q9UKQ2	Q9JLN6	15013428,26554003, 23643150	306,307,308	Annotated (existing reference for shedding information)
Disintegrin and metalloproteinase domain-containing protein 8 precursor	ADAM8	P78325	Q05910	12372841	309,310	Annotated (existing reference for shedding information)
Dystroglycan precursor	DAG1	Q14118	Q62165	19946898, 16701552	311	Annotated (existing reference for shedding information)
Ectodysplasin-A	EDA	Q92838	--	11309369	30	Annotated (existing reference for shedding information)
Endothelial protein C receptor precursor	PROCR	Q9UNN8	--	23774263	312	Annotated (existing reference for shedding information)
Endothelin B receptor precursor	EDNRB	P24530	--	12226103	313	Annotated (existing reference for shedding information)
Ephrin type-A receptor 4 precursor	Epha4	--	Q03137	25264256	46	Annotated (existing reference for shedding information)
Ephrin type-A receptor 7 precursor	Epha7	--	Q61772	22036564	314	Annotated (existing reference for shedding information)
Ephrin type-B receptor 1 precursor	EPHB1	P54762	--	12591933,	315	Annotated (existing reference for shedding information)
Ephrin type-B receptor 2 precursor	Ephb2	--	P54763	18713744	316	Annotated (existing reference for shedding information)
Ephrin-B1 precursor	EFNB1	P98172	--	17567680	317	Annotated (existing reference for shedding information)
Epidermal growth factor receptor precursor	EGFR	P00533	--	25719831, 23731208	318,319	Annotated (existing reference for shedding information)
Epithelial cell adhesion molecule precursor	EPCAM	P16422	--	19136966,24665393	59,60	Annotated (existing reference for shedding information)
Epithelial discoidin domain-containing receptor 1 precursor	DDR1	Q08345	--	16440311	320	Annotated (existing reference for shedding information)

E-selectin precursor	SELE	P16581	--	16565325,9441802	41,321	Annotated (existing reference for shedding information)
Fibroblast growth factor receptor 1 precursor	FGFR1	P11362	--	8692946,	322	Annotated (existing reference for shedding information)
Folate receptor alpha precursor	FOLR1	P15328	--	1846624	68	Annotated (existing reference for shedding information)
Fractalkine precursor	CX3CL1	P78423	O35188	16565325,11495925, 18951988	41,323,23	Annotated (existing reference for shedding information)
Furin precursor	FURIN	P09958	--	12220680,11237874	324,325	Annotated (existing reference for shedding information)
Gliomedin precursor	Gldn	Q8BMF8	--	17485493	326	Annotated (existing reference for shedding information)
Glutamate decarboxylase 2	Gad2	--	Q05683	16879709	327	Annotated (existing reference for shedding information)
Glutamate receptor 3 precursor	GRIA3	P42263	--	17202328	328	Annotated (existing reference for shedding information)
Glutamate receptor ionotropic, NMDA 1 precursor	Grin1	--	P35439	18629001,	329	Annotated (existing reference for shedding information)
Glypican-1 precursor	GPC1		P50593	20008810,	330	Annotated (existing reference for shedding information)
Glypican-3 precursor	GPC3	P51654	--	15665316,14610063	331,332	Annotated (existing reference for shedding information)
G-protein coupled receptor 126 precursor	GPR126	Q86SQ4	--	15189448	333	Annotated (existing reference for shedding information)
G-protein coupled receptor 56 precursor	GPR56	Q9Y653	--	25437104	334	Annotated (existing reference for shedding information)
Growth hormone receptor precursor	GHR	P10912	P19941	11309389	335	Annotated (existing reference for shedding information)
Hepatocyte growth factor precursor	HGF	P14210	--	8117273	336	Annotated (existing reference for shedding information)
Hepatocyte growth factor receptor precursor	MET	P08581	--	17875701,23746173	337,338	Annotated (existing reference for shedding information)
HLA class II histocompatibility antigen gamma chain	CD74	P04233	--	16107560	339	Annotated (existing reference for shedding information)
Insulin receptor precursor	INSR	P06213	--	17563065	340	Annotated (existing reference for shedding information)
Integral membrane protein 2B	ITM2B	Q9Y287	--	21170088,	234	Annotated (existing reference for shedding information)
Integrin alpha-4 precursor	ITGA4	P13612	--	1730718	341	Annotated (existing reference for shedding information)
Integrin alpha-6 precursor	ITGA6	P23229	--	17303120	342	Annotated (existing reference for shedding information)
Integrin alpha-V precursor	ITGAV	P06756	--	11741954,7505747	343,344	Annotated (existing reference for shedding information)
Integrin beta-1 precursor	ITGB1	P05556	--	22898815	345	Annotated (existing reference for shedding information)
Integrin beta-2 precursor	ITGB2	P05107	--	8692946,	322	Annotated (existing reference for shedding information)
Integrin beta-3 precursor	ITGB3	P05106	--	7505747	344	Annotated (existing reference for shedding information)

Integrin beta-4 precursor	ITGB4	P16144	A2A863	21750188	346	Annotated (existing reference for shedding information)
Intercellular adhesion molecule 1 precursor	ICAM1	P05362	--	16565325,16332693	41,347	Annotated (existing reference for shedding information)
Interferon alpha/beta receptor 2 precursor	IFNAR2	P48551	--	15286706	348	Annotated (existing reference for shedding information)
Interleukin-15 receptor subunit alpha precursor	IL15RA	Q13261	Q60819	16565325,15265897	41,349	Annotated (existing reference for shedding information)
Interleukin-18 precursor	IL18	Q14116	--	22678914	350	Annotated (existing reference for shedding information)
Interleukin-6 receptor subunit alpha precursor	IL6R	P08887	--	16565325	41	Annotated (existing reference for shedding information)
Junctional adhesion molecule A precursor	F11R	Q9Y624	--	18951988	23	Annotated (existing reference for shedding information)
Kit ligand precursor	KITLG	P21583	P20826	17344430	351	Annotated (existing reference for shedding information)
Klotho precursor	KL	Q9UEF7	--	25110992,19737556	70,71	Annotated (existing reference for shedding information)
Kunitz-type protease inhibitor 1 precursor	SPINT1	O43278	--	10544273	352	Annotated (existing reference for shedding information)
Leucyl-cystinyl aminopeptidase	LNPEP	Q9UIQ6	--	14751233	353	Annotated (existing reference for shedding information)
Leukocyte-associated immunoglobulin-like receptor 1 precursor	LAIR1	Q6GTX8	--	15350516	354	Annotated (existing reference for shedding information)
Leukosialin precursor	SPN	P16150	--	7507963, 7683406	355,356	Annotated (existing reference for shedding information)
Low affinity immunoglobulin epsilon Fc receptor	FCER2	P06734	P20693	19049889,26538900	193,357	Annotated (existing reference for shedding information)
Low-density lipoprotein receptor precursor	LDLR	P01130	--	3380796	358	Annotated (existing reference for shedding information)
Low-density lipoprotein receptor-related protein 1B precursor	LRP1B	Q9NZR2	Q9JI18	17227771	163	Annotated (existing reference for shedding information)
Low-density lipoprotein receptor-related protein 2 precursor	Lrp2	--	P98158	15180987	359	Annotated (existing reference for shedding information)
Low-density lipoprotein	LRP6	O75581	--	17326769	360	Annotated (existing reference for shedding information)

receptor-related protein 6 precursor						
L-selectin precursor	SELL	P14151	P18337	16565325,9812885	41,361	Annotated (existing reference for shedding information)
Ly6/PLAUR domain-containing protein 3 precursor	LYPD3	O95274	--	18979631	362	Annotated (existing reference for shedding information)
Lymphocyte activation gene 3 protein precursor	Lag3	--	Q61790	16565325,17245433	41,44	Annotated (existing reference for shedding information)
Macrophage colony-stimulating factor 1 precursor	CSF1	P09603	P07141	17982061	363	Annotated (existing reference for shedding information)
Major prion protein precursor	PRNP	P04156	--	11477090,18951988	364,23	Annotated (existing reference for shedding information)
Matrix metalloproteinase-14 precursor	MMP14	P50281	--	15137052	365	Annotated (existing reference for shedding information)
Mesothelin precursor	MSLN	Q13421	--	10500211, 16857795	366,367	Annotated (existing reference for shedding information)
Melanocyte protein PMEL precursor	PMEL	P40967	--	19047044,19884326	63,368	Annotated (existing reference for shedding information)
Membrane cofactor protein precursor	CD46	P15529	--	16735514,15307194	369,370	Annotated (existing reference for shedding information)
Meprin A subunit alpha precursor	MEP1A	Q16819	P28825	25617491, 9439598	371,238	Annotated (existing reference for shedding information)
Meprin A subunit beta precursor	MEP1B	Q16820	--	25617491, 12941954	371,372	Annotated (existing reference for shedding information)
MHC class I polypeptide-related sequence A precursor	MICA	Q29983	--	21389869,26161387,18676862	373,374,375	Annotated (existing reference for shedding information)
Monocyte differentiation antigen CD14 precursor	CD14	P08571	--	1880416	376	Annotated (existing reference for shedding information)
Mucin-1 precursor	MUC1	P15941	--	12441351, 15130087	377,378	Annotated (existing reference for shedding information)
Myelin basic protein	MBP	P02686	P02688	2600084	379	Annotated (existing reference for shedding information)
Myelin-associated glycoprotein precursor	MAG	P20916	--	18063113	380	Annotated (existing reference for shedding information)
Nectin-1 precursor	PVRL1	Q15223	Q15223	22118475,20501653,24665393	381,382,60	Annotated (existing reference for shedding information)
Natural cytotoxicity triggering receptor 3 ligand 1 precursor	NCR3LG1	Q68D85	--	24780758	383	Annotated (existing reference for shedding information)
Neogenin precursor	Neo1	--	P97798	26651291, 21645559	384,47	Annotated (existing reference for shedding information)
Neprilysin	MME	P08473	--	24495806	385	Annotated (existing reference for shedding information)
Netrin receptor DCC precursor	Dcc	--	Q63155	10958786	386	Annotated (existing reference for shedding information)

Neural cell adhesion molecule L1 precursor	L1CAM	P32004	P11627	11706054,18951988	387,23	Annotated (existing reference for shedding information)
Neural cell adhesion molecule L1-like protein precursor	Chl1	--	P70232	14761956	388	Annotated (existing reference for shedding information)
Neurexin-3-beta precursor	NRXN3	Q9HDB5	--	17901375	143	Annotated (existing reference for shedding information)
Neurogenic locus notch homolog protein 1 precursor	NOTCH1	P46531	Q01705	10882063,18951988	389,23	Annotated (existing reference for shedding information)
Neurogenic locus notch homolog protein 2 precursor	NOTCH2	Q04721	O35516	10958687,24842903	390,391	Annotated (existing reference for shedding information)
Neurogenic locus notch homolog protein 3 precursor	NOTCH3	Q9UM47	--	24842903	391	Annotated (existing reference for shedding information)
Neuronal pentraxin receptor	Nptxr	--	Q99J85	18367087	392	Annotated (existing reference for shedding information)
Neuropilin-2 precursor	NRP2	O60462	--	19790074	24	Annotated (existing reference for shedding information)
Occludin	OCLN	Q16625	--	17038551,15472219,24665393	393,394,60	Annotated (existing reference for shedding information)
Opioid-binding protein/cell adhesion molecule precursor	Opcml	--	P32736	25538237	84	Annotated (existing reference for shedding information)
Oxidized low-density lipoprotein receptor 1	OLR1	P78380	--	16565325,16061745	41,395	Annotated (existing reference for shedding information)
Parathyroid hormone/parathyroid hormone-related peptide receptor precursor	PTH1R	Q03431	--	20080964	94	Annotated (existing reference for shedding information)
Platelet endothelial cell adhesion molecule precursor	PECAM1	P16284	--	16565325, 16507710	41,396	Annotated (existing reference for shedding information)
Platelet glycoprotein Ib alpha chain precursor	GP1BA	P07359	O35930	24119228, 19820200	53,397	Annotated (existing reference for shedding information)
Platelet glycoprotein V precursor	GP5	P40197	O08742	18951988	23	Annotated (existing reference for shedding information)
Platelet glycoprotein VI precursor	GP6	Q9HCN6	--	24674813,17445093	398,399	Annotated (existing reference for shedding information)
Platelet receptor Gi24 precursor	C10orf54	Q9H7M9	--	20666777	400	Annotated (existing reference for shedding information)
Polycystin-1 precursor	PKD1	P98161	--	17525154	401	Annotated (existing reference for shedding information)
Probetacellulin precursor	BTC	P35070	Q05928	14993236,11823465,18951988	54,55,23	Annotated (existing reference for shedding information)
Pro-epidermal growth factor	EGF	P01133	P01132	14993236,18951988	54,23	Annotated (existing reference for shedding information)

precursor						
Proepiregulin precursor	EREG	O14944	Q61521	19049889,18951988	193,23	Annotated (existing reference for shedding information)
Proheparin-binding EGF-like growth factor precursor	HBEGF	Q99075	Q06186	11038170,14985295, 11786904, 11825873, 18951988	402,403,404,405,23	Annotated (existing reference for shedding information)
Prolow-density lipoprotein receptor-related protein 1 precursor	LRP1	Q07954	--	19371428	406	Annotated (existing reference for shedding information)
Pro-neuregulin-1, membrane-bound isoform precursor	NRG1	Q02297	Q6DR99	18951988,20215529	23,407	Annotated (existing reference for shedding information)
Protein deglycase DJ-1 precursor	PARK7	Q99497	--	20969476	408	Annotated (existing reference for shedding information)
Proteinase-activated receptor 1 precursor	F2R	P25116	--	14982936	409	Annotated (existing reference for shedding information)
Proteinase-activated receptor 2 precursor	F2RL1	P55085	--	12594060	410	Annotated (existing reference for shedding information)
Protransforming growth factor alpha precursor	TGFA	P01135	P48030	10773880, 26658844,12590602	411,412,413	Annotated (existing reference for shedding information)
P-selectin glycoprotein ligand 1 precursor	SELPLG	Q14242	Q62170	14507929,25511460	414,415	Annotated (existing reference for shedding information)
Receptor tyrosine-protein kinase erbB-2 precursor	ERBB2	P04626	--	16627989,21549508	416,417	Annotated (existing reference for shedding information)
Receptor tyrosine-protein kinase erbB-4 precursor	ERBB4	Q15303	--	12475204	3	Annotated (existing reference for shedding information)
Receptor-type tyrosine-protein phosphatase kappa precursor	PTPRK	Q15262	P35822	16404719	418	Annotated (existing reference for shedding information)
Receptor-type tyrosine-protein phosphatase mu precursor	PTPRM	P28827	--	8620001,19690139	419,420	Annotated (existing reference for shedding information)
Reversion-inducing cysteine-rich protein with Kazal motifs precursor	RECK	O95980	--	19022775	421	Annotated (existing reference for shedding information)
Scavenger receptor cysteine-rich type 1 protein M130 precursor	CD163	Q86VB7	Q2VLH6	20807704	422	Annotated (existing reference for shedding information)
Semaphorin-4B precursor	Sema4b	--	Q62179	23430253	72	Annotated (existing reference for shedding information)
Semaphorin-4C precursor	SEMA4C	Q9C0C4	--	20041192	132	Annotated (existing reference for shedding information)

Sodium- and chloride-dependent glycine transporter 2	Slc6a5	--	P58295	14675166	423	Annotated (existing reference for shedding information)
Sodium channel subunit beta-1 precursor	Scn1b	--	P97952	15824102,18694383	424,425	Annotated (existing reference for shedding information)
Sodium channel subunit beta-2 precursor	Scn2b	--	Q56A07	15824102,18694383	424,425	Annotated (existing reference for shedding information)
Sodium channel subunit beta-3 precursor	Scn3b	--	Q8BHK2	15824102,18694383	424,425	Annotated (existing reference for shedding information)
Sodium channel subunit beta-4 precursor	Scn4b	--	Q7M729	15824102,18694383	424,425	Annotated (existing reference for shedding information)
Sortilin-related receptor precursor	SORL1	Q92673	--	16393139, 24699135,25443876,20047743,22541650	426,33,34,35,36	Annotated (existing reference for shedding information)
Sonic hedgehog protein precursor	SHH	Q15465	--	23118222	58	Annotated (existing reference for shedding information)
Stromelysin-1 precursor	MMP3	P08254	--	14681236	427	Annotated (existing reference for shedding information)
Suppressor of tumorigenicity 14 protein	ST14	Q9Y5Y6	--	11231297	428	Annotated (existing reference for shedding information)
Syndecan-1 precursor	SDC1	P18827	P18828	25321193, 12904296	20,429	Annotated (existing reference for shedding information)
Syndecan-3	SDC3	O75056	P33671	12929127,14504279	430,431	Annotated (existing reference for shedding information)
Syndecan-4 precursor	SDC4	P31431	--	26261514,	25	Annotated (existing reference for shedding information)
T-cell surface glycoprotein CD4 precursor	CD4	P01730	--	7722333, 23700441	207,432	Annotated (existing reference for shedding information)
Thy-1 membrane glycoprotein precursor	THY1	P04216	--	77877,2900838,16049324	433,434,435	Annotated (existing reference for shedding information)
Tissue factor pathway inhibitor precursor	TFPI	P10646	--	10859319	436	Annotated (existing reference for shedding information)
Tomoregulin-2 precursor	TMEFF2	Q9UIK5	--	17942404	437	Annotated (existing reference for shedding information)
Transmembrane glycoprotein NMB precursor	GPNMB	Q14956	--	20711474,20056711	438,439	Annotated (existing reference for shedding information)
Transmembrane protease serine 2 precursor	TMPRSS2	O15393	--	11245484	440	Annotated (existing reference for shedding information)
Tumor necrosis factor ligand	TNFSF11	O14788	O35235	10224132,17018528	441,442	Annotated (existing reference for shedding information)

superfamily member 11						
Tumor necrosis factor ligand superfamily member 15	TNFSF15	O95150	--	21378310,20675618	443,444	Annotated (existing reference for shedding information)
Tumor necrosis factor ligand superfamily member 6	FASLG	P48023	P41047	16565325,11212252	41,445	Annotated (existing reference for shedding information)
Tumor necrosis factor ligand superfamily member 8	TNFSF8	P32971	--	12777399,24447865	446,447	Annotated (existing reference for shedding information)
Tumor necrosis factor precursor	TNF	P01375	P06804	16565325	41	Annotated (existing reference for shedding information)
Tumor necrosis factor receptor superfamily member 16 precursor	NGFR	P08138	P07174	14638693,17904861	56,57	Annotated (existing reference for shedding information)
Tumor necrosis factor receptor superfamily member 1A precursor	TNFRSF1A	P19438	P25118	16565325	41	Annotated (existing reference for shedding information)
Tumor necrosis factor receptor superfamily member 1B precursor	TNFRSF1B	P20333	--	16565325	41	Annotated (existing reference for shedding information)
Tumor necrosis factor receptor superfamily member 5 precursor	CD40	P25942	--	16565325,12810728	41,448	Annotated (existing reference for shedding information)
Tumor necrosis factor receptor superfamily member 6 precursor	FAS	P25445	--	15077180	131	Annotated (existing reference for shedding information)
Urokinase plasminogen activator surface receptor precursor	PLAUR	Q03405	P35456	11342439	449	Annotated (existing reference for shedding information)
Vascular endothelial growth factor A precursor	VEGFA	P15692	--	24501421	450	Annotated (existing reference for shedding information)
Very low-density lipoprotein receptor precursor	VLDLR	P98155	--	15950758	165	Annotated (existing reference for shedding information)

Table S2. The details of the shed membrane protein members in each group of function category

Function categories	Categories note	Protein Name	Gene Symbol	UniProtID (human)	UniProtID (other species)	PubMedID	Reference
disease	acute renal failure (ARF)	Toll-like receptor 4 precursor	Tlr4	--	Q9QUK6	19864413	282
	Alzheimer Disease	Calsyntenin-2 precursor	CLSTN2	Q9H4D0	--	11927537	293
	Alzheimer Disease	Collagen alpha-1(XXV) chain	COL25A1	Q9BXS0	--	15259011	107
	Alzheimer Disease	Calsyntenin-3 precursor	CLSTN3	Q9BQT9	--	26261514	25
	Alzheimer disease	Amyloid beta A4 protein precursor	APP	P05067	P12023	19864413, 11161476	282,283
	Alzheimer Disease	Calsyntenin-1 precursor	CLSTN1	O94985	Q9DDD3	15086406	211
	atrial fibrillation (AF)	Syndecan-4 precursor	SDC4	P31431	--	25205731	83
	autoimmune disease	T-cell-specific surface glycoprotein CD28 precursor	CD28	P10747	--	10500211, 16857795	366,367
	autoimmunity	Carcinoembryonic antigen-related cell adhesion molecule 1 precursor	CEACAM1	P13688	--	19049889,15692570, 18951988, 16998833	193,278,23,279
	cancer	CD226 antigen precursor	CD226	Q15762	--	19490613	239
	cancer	Suppressor of tumorigenicity 14 protein homolog	St14	--	P56677	25245289, 20652801	86,87
	cancer	Receptor-binding cancer antigen expressed on SiSo cells	EBAG9	O00559	--	25177692	88
	cancer	CUB domain-containing protein 1 precursor	CDCP1	Q9H5V8	--	20551327	227
	cancer	Glypican-3 precursor	GPC3	P51654	--	15665316, 14610063	331,332
	cancer	Ly6/PLAUR domain-containing protein 3 precursor	LYPD3	O95274	--	18979631	362
	cancer	Aminopeptidase N	ANPEP	P15144	--	12473585	99
	cancer (AML)	Myeloid cell surface antigen CD33 precursor	CD33	P20138	--	24674885, 20362641, 16828866	175,176,177
	cancer (breast cancer)	Nectin-4 precursor	PVRL4	Q96NY8	--	15784625	29
	cancer (breast cancer)	Transmembrane glycoprotein NMB precursor	GPNMB	Q14956	--	20711474, 20056711	438,439
	cancer (breast)	Receptor tyrosine-protein kinase erbB-2 precursor	ERBB2	P04626	--	16627989, 21549508	416,417
	cancer (chronic lymphocytic leukemia)	OX-2 membrane glycoprotein precursor	CD200	P41217	--	11309369, 22875025	30,31
	cancer (colon cancer)	Cadherin-17 precursor	CDH17	Q12864	--	25336636	32
	cancer (Gastrointestinal)	Amphiregulin precursor	AREG	P15514	P31955	24520077, 16769815, 15337756	263,261,262
	cancer (hepatocellular carcinoma)	Roundabout homolog 1 precursor	ROBO1	Q9Y6N7	--	16740745	201
	cancer (hepatocellular carcinoma)	Basal cell adhesion molecule precursor	BCAM	P50895	--	25051049	272
	cancer (invasion)	Annexin A2	ANXA2	P07355	--	18721140	270
	cancer (invasion)	Protein jagged-1 precursor	JAG1	P78504	--	22936788	16

cancer (invasion)	Syndecan-1 precursor	SDC1	P18827	P18828	25321193, 12904296	20,429
cancer (invasion,breast,prostate,gastric carcinoma.)	Cadherin-1 precursor	CDH1	P12830	Q9R0T4	22936788, 25967040, 24712279	16,17,18
cancer (leukaemia)	CD9 antigen	CD9	P21926	--	1390240, 8180600	108,109
cancer (lung metastasis)	Ephrin-A1 precursor	EFNA1	P20827	--	23686306	19
cancer (ovarian cancer)	Mucin-16	MUC16	Q8WXI7	--	16885150	217
cancer (ovarian carcinoma, mesothelioma)	Mesothelin precursor	MSLN	Q13421	--	22553493, 18951988	22,23
cancer (pancreatic cancer)	Protocadherin Fat 1 precursor	FAT1	Q14517	--	15922730, 24625754	191,192
cancer (prostate cancer)	Tomoregulin-2 precursor	TMEFF2	Q9UIK5	--	17942404	437
cancer (prostate cancer)	Transmembrane protease serine 2 precursor	TMPRSS2	O15393	--	11245484	440
cancer (prostate)	Cadherin-2 precursor	CDH2	P19022	P15116	19864413	282
cancer (renal carcinoma)	Carbonic anhydrase 9 precursor	CA9	Q16790	--	9439598	238
cancer (tumor escape targeting by NK and immuno therapy)	Natural cytotoxicity triggering receptor 3 ligand 1 precursor	NCR3LG1	Q68D85	--	24780758	383
cancer (tumor escape targeting by NK and immuno therapy)	MHC class I polypeptide-related sequence B precursor	MICB	Q29980	--	16698441	174
cancer (tumor escape targeting by NK and immuno therapy)	NKG2D ligand 2 precursor	ULBP2	Q9BZM5	--	16510567	183
cancer (tumor escape targeting by NK and immuno therapy)	MHC class I polypeptide-related sequence A precursor	MICA	Q29983	--	21389869, 26161387, 18676862	373,374,375
cancer (tumor escape)	CD83 antigen precursor	CD83	Q01151	--	18513799	241
cancer (tumor escape)	Tumor necrosis factor receptor superfamily member 6 precursor	FAS	P25445	--	15077180	131
cancer (tumor invasion)	Cadherin-3 precursor	CDH3	P22223	--	19901964	280
cancer (tumor invasion)	Platelet receptor Gi24 precursor	C10orf54	Q9H7M9	--	20666777	400
cancer (tumor invasion)	Receptor-type tyrosine-protein phosphatase kappa precursor	PTPRK	Q15262	P35822	16404719	418
cancer (tumor invasion)	Inactive tyrosine-protein kinase 7 precursor	PTK7	Q13308	--	23095747	247
cancer (tumor invasion)	40S ribosomal protein SA	RPSA	P08865	--	8655633	187
cancer (tumor suppress)	Transforming growth factor beta receptor type 3 precursor	TGFBR3	Q03167	--	24966170	221
cancer (urothelial bladder cancer)	Epidermal growth factor receptor precursor	EGFR	P00533	--	25719831, 23731208	318,319
cancer (chronic lymphocytic leukemia)	CAMPATH-1 antigen precursor	CD52	P31358	--	15329909	106
cancer (glioma)	Leucine-rich repeats and immunoglobulin-like domains protein	LRIG2	O94898	--	20041192, 25353163	132,159

		2 precursor					
	cardiac hypertrophy	Proheparin-binding EGF-like growth factor precursor	HBEGF	Q99075	Q06186	11786904, 11825873, 18951988,11038170, 14985295	404,405,23,402,403
	cardiovascular disease	Sortilin-related receptor precursor	SORL1	Q92673	--	16393139	426
	Creutzfeldt-Jakob disease	Major prion protein precursor	PRNP	P04156	--	11477090, 18951988	364,23
	cystic fibrosis	T-cell surface antigen CD2 precursor	CD2	P06729	--	7722333	207
	cystic fibrosis	T-cell surface glycoprotein CD8 alpha chain precursor	CD8A	P01732	--	6203997, 6193233, 7722333	209,210,207
	cystic fibrosis, Rheumatoid Arthritis	T-cell surface glycoprotein CD4 precursor	CD4	P01730	--	7722333, 23700441	207,432
	deficiency anemia (IRIDA)	Transmembrane protease serine 6	TMPRSS6	Q8IU80	--	24867957	90
	diabetes	Insulin receptor precursor	INSR	P06213	--	17563065	340
	diabetes mellitus (DM)	C-type lectin domain family 1 member B	CLEC1B	Q9P126	--	26290605	37
	epithelial erosion	Integrin beta-4 precursor	ITGB4	P16144	A2A863	21750188	346
	gastric mucosa Infection by H. pylori	Protransforming growth factor alpha precursor	TGFA	P01135	P48030	26658844,12590602, 12590602	412,413,413
	hepatocyte damage	Receptor-type tyrosine-protein phosphatase gamma precursor	PTPRG	P23470	--	25775014	40
	idiopathic interstitial pneumonia (IIP)	Cell adhesion molecule 1 precursor	CADM1	Q9BY67	Q8R5M8	26231557, 24964098, 22172944	27,28,289
	infection (HIV)	Angiotensin-converting enzyme 2 precursor	ACE2	Q9BYF1	--	17150042,15983030, 18490652	246,267,268
	infection (HIV)	Hepatitis A virus cellular receptor 2 precursor	HAVCR2	Q8TDQ0	--	25609823	26
	infection (HIV)	Platelet endothelial cell adhesion molecule precursor	PECAM1	P16284	--	16507710, 16565325	396,41
	ischaemia	BDNF/NT-3 growth factors receptor precursor	Ntrk2	--	P15209	26712630	101
	neurodegenerative disorders, dementia	Platelet-derived growth factor receptor beta precursor	PDGFRB	P09619	P05622	20529858, 26407747	38,39
	Parkinson disease (PD)	Alpha-synuclein	SNCA	P37840	--	15863497	21
	Parkinson's disease	Prosaposin receptor GPR37 precursor	GPR37	O15354	--	26869225	190
	Parkinson's disease	Protein deglycase DJ-1 precursor	PARK7	Q99497	--	20969476	408
	Polycystic Kidney And Hepatic Disease	Fibrocystin precursor	PKHD1	P08F94	--	16956880, 17470460	129,130
	preeclampsia	Nephrin precursor	NPHS1	O60500	--	18287402	178
	rheumatoid arthritis	Neuropilin-2 precursor	NRP2	O60462	--	19790074	24
	thrombus formation	Platelet glycoprotein Ib alpha chain precursor	GP1BA	P07359	O35930	19820200, 24119228	397,53
immune and inflammation	cell adhesion molecule	Annexin A1	ANXA1	P04083	--	17023068	269
	cell adhesion molecule	CD44 antigen precursor	CD44	P16070	P15379	16565325, 15197174, 18951988	41,287,23
	cell adhesion molecule	E-selectin precursor	SELE	P16581	--	16565325, 9441802	41,321
	cell adhesion molecule	Intercellular adhesion molecule 1 precursor	ICAM1	P05362	--	16565325, 16332693	41,347

cell adhesion molecule	Intercellular adhesion molecule 2 precursor	ICAM2	P13598	--	19524015	141
cell adhesion molecule	Junctional adhesion molecule B precursor	JAM2	P57087	--	25367033	249
cell adhesion molecule	L-selectin precursor	SELL	P14151	P18337	16565325, 9812885	41,361
cell adhesion molecule	P-selectin precursor	Selp	--	Q01102	16565325	41
cell adhesion molecule	Vascular cell adhesion protein 1 precursor	VCAM1	P19320	P29533	16565325, 15949468	41,254
cell adhesion molecule	Cadherin-5 precursor	CDH5	P33151	--	16565325, 18951988	41,23
cell adhesion molecule	Junctional adhesion molecule A precursor	F11R	Q9Y624	--	18951988	23
cell adhesion molecule	Membrane primary amine oxidase	AOC3	Q16853	--	22618595	173
cell adhesion molecule	CD166 antigen precursor	ALCAM	Q13740	--	18171982	285
cell adhesion molecule	Cadherin-11 precursor	CDH11	P55287	--	26312857	62
cell adhesion molecule	Cell surface glycoprotein MUC18 precursor	MCAM	P43121	--	19229070	116
cell adhesion molecule	Intercellular adhesion molecule 3 precursor	ICAM3	P32942	--	7876564	142
cell adhesion molecule	Coxsackievirus and adenovirus receptor homolog precursor	Cxadr	--	P97792	24015300	296
cell receptor	CD160 antigen precursor	CD160	O95971	--	17237375	110
cell receptor	CD27 antigen precursor	CD27	P26842	--	1311261	286
cell receptor	T-cell surface glycoprotein CD5 precursor	CD5	P06127	--	10488739	208
cell receptor	SLAM family member 5 precursor	Cd84	--	Q18PI6	23025437	203
cell receptor	T-lymphocyte activation antigen CD86 precursor	CD86	P42081	--	11021528	214
cell receptor	Collectin-12	COLEC12	Q5KU26	--	26290605	37
cell receptor	Tumor necrosis factor receptor superfamily member 21 precursor	TNFRSF21	O75509	--	15118097	226
cell receptor	Integrin alpha-6 precursor	ITGA6	P23229	--	17303120	342
cell receptor	Integrin alpha-V precursor	ITGAV	P06756	--	11741954, 7505747	343,344
cell receptor	Integrin beta-1 precursor	ITGB1	P05556	--	22898815	345
cell receptor	Integrin beta-3 precursor	ITGB3	P05106	--	7505747	344
cell receptor	Oxidized low-density lipoprotein receptor 1	OLR1	P78380	--	16565325, 16061745	41,395
cell receptor	Tyrosine-protein kinase Mer precursor	MERTK	Q12866	--	26316303	228
cell receptor	Polymeric immunoglobulin receptor precursor	PIGR	P01833	--	3108385	250
cell receptor	Tumor necrosis factor receptor superfamily member 13B	TNFRSF13B	O14836	--	25505277	92
cell receptor	Toll-like receptor 9 precursor	TLR9	Q9NR96	Q9EQU3	18820679, 21604257	218,219
cell receptor	Tumor necrosis factor receptor superfamily member 27	EDA2R	Q9HAV5	--	20501644	93

cell receptor	Scavenger receptor cysteine-rich type 1 protein M130 precursor	CD163	Q86VB7	Q2VLH6	20807704	422
cell receptor	Leukocyte-associated immunoglobulin-like receptor 1 precursor	LAIR1	Q6GTX8	--	15350516	354
cell receptor	Advanced glycosylation end product-specific receptor precursor	AGER	Q15109	--	18952609	98
cell receptor	Complement component 1 Q subcomponent-binding protein, mitochondrial precursor	C1QBP	Q07021	--	11773076	294
cell receptor	Dipeptidyl peptidase 4	DPP4	P27487	P28843	8878393, 25217834	120,121
cell receptor	Immunoglobulin alpha Fc receptor precursor	FCAR	P24071	--	20059578	140
cell receptor	Macrophage mannose receptor 1 precursor	MRC1	P22897	Q61830	10545481, 9722572	169,170
cell receptor	Complement receptor type 2 precursor	CR2	P20023	--	12938215	45
cell receptor	Complement receptor type 1 precursor	CR1	P17927	--	7957565	119
cell receptor	Integrin alpha-4 precursor	ITGA4	P13612	--	1730718	341
cell receptor	Low affinity immunoglobulin gamma Fc region receptor II-a precursor	FCGR2A	P12318	--	8254192	160
cell receptor	Low affinity immunoglobulin gamma Fc region receptor III-A precursor	FCGR3A	P08637	--	23487023	161
cell receptor	Monocyte differentiation antigen CD14 precursor	CD14	P08571	--	1880416	376
cell receptor	Low affinity immunoglobulin epsilon Fc receptor	FCER2	P06734	P20693	19049889, 26538900	193,357
cell receptor	Low affinity immunoglobulin gamma Fc region receptor III-B precursor	FCGR3B	O75015	--	23228566	162
cell receptor	Toll-like receptor 2 precursor	TLR2	O60603	--	25531754	215
cell receptor	Toll-like receptor 3 precursor	Tlr3	--	Q99MB1	25305318	216
cell receptor	Lymphocyte activation gene 3 protein precursor	Lag3	--	Q61790	16565325, 17245433	41,44
cell receptor	Platelet glycoprotein 4	Cd36	--	Q08857	8655633	187
cell receptor	T-cell immunoglobulin and mucin domain-containing protein 2 precursor	Timd2	--	Q8R183	24164679	206
cell receptor	T-cell immunoglobulin and mucin domain-containing protein 4 precursor	Timd4	--	Q6U7R4	24286866	136
cell receptor	Triggering receptor expressed on myeloid cells 1 precursor	TREM1	Q9NP99	--	17785845	223
cell receptor	Triggering receptor expressed on myeloid cells 2 precursor	TREM2	Q9NZC2	--	24078628	224
cell receptor	Vesicular integral-membrane protein VIP36 precursor	LMAN2	Q12907	--	22016386	255
cell receptor	Lymphatic vessel endothelial hyaluronic acid receptor 1	LYVE1	Q9Y5Y7	--	26966180	166

	precursor					
cell receptor	Hepatitis A virus cellular receptor 1 precursor	HAVCR1	Q96D42	Q5QNS5	22843853, 24286866	135,136
cell receptor	Trem-like transcript 1 protein precursor	TREML1	Q86YW5	--	16505478	222
cell receptor (EPCR)	Endothelial protein C receptor precursor	PROCR	Q9UNN8	--	23774263	312
cell receptor (GPCR)	Adhesion G protein-coupled receptor E2 precursor	ADGRE2	Q9UHX3	--	16982628, 12860403	133,258
cell receptor (GPCR)	CD97 antigen precursor	CD97	P48960	Q9Z0M6	15576472, 23447688	114,115
cell surface protein	Membrane cofactor protein precursor	CD46	P15529	--	16735514, 15307194	369,370
cell surface protein	HLA class I histocompatibility antigen, alpha chain E precursor	HLA-E	P13747	--	16920947	138
cell surface protein	HLA class I histocompatibility antigen, alpha chain G precursor	HLA-G	P17693	--	14697234	139
cell surface protein	Thy-1 membrane glycoprotein precursor	THY1	P04216	--	77877, 2900838, 16049324	433,434,435
cell surface protein	Leukosialin precursor	SPN	P16150	--	7507963, 7683406	355,356
cell surface protein	Complement decay-accelerating factor precursor	CD55	P08174	--	2477368	295
cell surface protein	HLA class I histocompatibility antigen, A-2 alpha chain precursor	HLA-A	P01892	--	17150042	246
complement inhibitor	CD59 glycoprotein precursor	CD59	P13987	--	7558140	288
cytokine	Macrophage colony-stimulating factor 1 precursor	CSF1	P09603	P07141	17982061	363
cytokine	C-X-C motif chemokine 16 precursor	CXCL16	Q9H2A7	Q8BSU2	16565325, 23428418, 18951988	41,42,23
cytokine	Tumor necrosis factor ligand superfamily member 6	FASLG	P48023	P41047	16565325, 11212252	41,445
cytokine	Tumor necrosis factor precursor	TNF	P01375	P06804	16565325	41
cytokine	Tumor necrosis factor ligand superfamily member 11	TNFSF11	O14788	O35235	10224132, 17018528	441,442
cytokine	Fractalkine precursor	CX3CL1	P78423	O35188	16565325, 11495925, 18951988	41,323,23
cytokine	Tumor necrosis factor ligand superfamily member 9	TNFSF9	P41273	--	11564827	91
cytokine	Tumor necrosis factor ligand superfamily member 8	TNFSF8	P32971	--	12777399, 24447865	446,447
cytokine	CD40 ligand	CD40LG	P29965	--	18951988	23
cytokine	Interleukin-18 precursor	IL18	Q14116	--	22678914	350
cytokine receptor	Tumor necrosis factor receptor superfamily member 8 precursor	TNFRSF8	P28908	--	16565325, 11120787	41,43
cytokine receptor	Tumor necrosis factor receptor superfamily member 5 precursor	CD40	P25942	--	16565325, 12810728	41,448
cytokine receptor	Complement component C1q receptor precursor	CD93	Q9NPY3	--	16565325, 16002728	41,118
cytokine receptor	Granulocyte-macrophage colony-stimulating factor receptor subunit alpha precursor	CSF2RA	P15509	--	16565325	41
cytokine receptor	Interferon alpha/beta receptor 2 precursor	IFNAR2	P48551	--	15286706	348

	cytokine receptor	Interleukin-11 receptor subunit alpha precursor	IL11RA	Q14626	--	26876177	145
	cytokine receptor	Interleukin-13 receptor subunit alpha-2 precursor	IL13RA2	Q14627	--	17603012, 18694590	146,147
	cytokine receptor	Interleukin-15 receptor subunit alpha precursor	IL15RA	Q13261	Q60819	16565325, 15265897	41,349
	cytokine receptor	Interleukin-1 receptor type 2 precursor	IL1R2	P27930	P27931	16565325, 10210771	41,144
	cytokine receptor	Interleukin-23 receptor precursor	IL23R	Q5VWK5	--	26961870	149
	cytokine receptor	Interleukin-4 receptor subunit alpha precursor	IL4R	P24394	--	10341317	150
	cytokine receptor	Interleukin-5 receptor subunit alpha precursor	IL5RA	Q01344	--	12444155	151
	cytokine receptor	Interleukin-6 receptor subunit alpha precursor	IL6R	P08887	--	16565325	41
	cytokine receptor	Tumor necrosis factor receptor superfamily member 1A precursor	TNFRSF1A	P19438	P25118	16565325	41
	cytokine receptor	Tumor necrosis factor receptor superfamily member 1B precursor	TNFRSF1B	P20333	--	16565325	41
	cytokine receptor	Leptin receptor precursor	LEPR	P48357	--	11564702	155
	cytokine receptor	Interleukin-6 receptor subunit beta precursor	IL6ST	P40189	--	8353278	152
	cytokine receptor	Interleukin-7 receptor subunit alpha precursor	IL7R	P16871	--	17956896	153
	cytokine receptor	Interleukin-2 receptor subunit beta	IL2RB	P14784	--	16565325, 20495002	41,148
	cytokine receptor	Macrophage colony-stimulating factor 1 receptor precursor	CSF1R	P07333	P09581	11160199	168
	cytokine receptor	Integrin beta-2 precursor	ITGB2	P05107	--	8692946	322
	cytokine receptor	HLA class II histocompatibility antigen gamma chain	CD74	P04233	--	16107560	339
	cytokine receptor	Tumor necrosis factor ligand superfamily member 15	TNFSF15	O95150	--	21378310, 20675618	443,444
	cytokine receptor	Cytokine receptor common subunit gamma precursor	Il2rg	--	P34902	11133759	300
	cytokine receptor	Tumor necrosis factor receptor superfamily member 11A precursor	Tnfrsf11a	--	O35305	20118276	225
	ligand	CD48 antigen precursor	CD48	P09326	--	20833258, 9418191, 11513145	111,112,113
	ligand	PILR alpha-associated neural protein precursor	Pianp	--	Q6P1B3	27349870, 26188512	185,186
	ligand	V-set domain-containing T-cell activation inhibitor 1 precursor	VTCN1	Q7Z7D3	--	24848066	235
	ligand	CD276 antigen precursor	CD276	Q5ZPR3	--	18194267	240
	ligand	P-selectin glycoprotein ligand 1 precursor	SELPLG	Q14242	Q62170	14507929, 25511460	414,415
	ligand	Lymphocyte function-associated antigen 3 precursor	CD58	P19256	--	9732704	167
	ligand	Fms-related tyrosine kinase 3 ligand precursor	Flt3lg	--	P49772	15077180	131
central nervous system	--	Contactin-2 precursor	Cntn2	--	Q61330	22728825	74

(neuron)

	--	Seizure 6-like protein precursor	Sez6l	--	Q6P1D5	23430253	72
	--	Seizure protein 6 precursor	Sez6	--	Q7TSK2	22728825	74
	cell adhesion molecule	Leucine-rich repeat transmembrane protein FLRT1 precursor	FLRT1	Q9NZU1	--	21673655	156
	cell adhesion molecule	Leucine-rich repeat transmembrane protein FLRT2 precursor	FLRT2	O43155	--	21673655	156
	cell adhesion molecule	Leucine-rich repeat transmembrane protein FLRT3 precursor	FLRT3	Q9NZU0	--	21673655	156
	cell adhesion molecule	Neural cell adhesion molecule 1 precursor	NCAM1	P13591	--	15884014	48
	cell adhesion molecule	Nectin-1 precursor	PVRL1	Q15223	Q15223	22118475, 20501653, 24665393	381,382,60
	cell adhesion molecule	Neurexin-3-beta precursor	NRXN3	Q9HDB5	--	17901375	143
	cell adhesion molecule	Neuronal cell adhesion molecule precursor	NRCAM	Q92823	--	16357171	181
	cell adhesion molecule	Protocadherin gamma-B4 precursor	PCDHGB4	Q9UN71	--	19049889, 16751190	193,194
	cell adhesion molecule	Protocadherin gamma-C3 precursor	PCDHGC3	Q9UN70	--	19049889, 16751190	193,194
	cell adhesion molecule	Neural cell adhesion molecule L1 precursor	L1CAM	P32004	P11627	11706054, 18951988	387,23
	cell adhesion molecule	Myelin-associated glycoprotein precursor	MAG	P20916	--	18063113	380
	cell adhesion molecule	Down syndrome cell adhesion molecule homolog precursor	Dscam	--	Q9ERC8	23300735	122
	cell adhesion molecule	Neuroligin-1 precursor	Nlgn1	--	Q99K10	23083742	180
	cell adhesion molecule	Intercellular adhesion molecule 5 precursor	Icam5	--	Q60625	17901375	143
	cell adhesion molecule	Neurofascin precursor	Nfasc	--	P97685	16360652	179
	cell adhesion molecule	Neural cell adhesion molecule L1-like protein precursor	Chl1	--	P70232	14761956	388
	cell adhesion molecule	Ninjurin-1	Ninj1	--	O70131	23142597	85
	cell adhesion molecule (IgLON)	Neuronal growth regulator 1 precursor	Negr1	--	Q9Z0J8	25538237	84
	cell adhesion molecule (IgLON)	Limbic system-associated membrane protein precursor	Lsamp	--	Q62813	25538237	84
	cell adhesion molecule (IgLON)	Neurotrimin precursor	Ntm	--	Q62718	25538237	84
	cell adhesion molecule (IgLON)	Opioid-binding protein/cell adhesion molecule precursor	Opcml	--	P32736	25538237	84
	cell receptor	Neogenin precursor	Neo1	--	P97798	26651291, 21645559	384,47
	cell receptor	Netrin receptor DCC precursor	Dcc	--	Q63155	10958786	386
	cell receptor	Glutamate receptor ionotropic, NMDA 1 precursor	Grin1	--	P35439	18629001	329
	cell receptor	Neurogenic locus notch homolog protein 1 precursor	NOTCH1	P46531	Q01705	10882063, 18951988	389,23
	cell receptor	Neurogenic locus notch homolog protein 2 precursor	NOTCH2	Q04721	O35516	10958687, 24842903	390,391
	cell receptor	Neurogenic locus notch homolog protein 3 precursor	NOTCH3	Q9UM47	--	24842903	391
	cell receptor	Semaphorin-4C precursor	SEMA4C	Q9C0C4	--	20041192	132

cell receptor	Reticulon-4 receptor precursor	RTN4R	Q9BZR6	Q99M75	15331667, 16849393	49,50
cell receptor	Glutamate receptor 3 precursor	GRIA3	P42263	--	17202328	328
cell receptor	Neuronal pentraxin receptor	Nptxr	--	Q99J85	18367087	392
cell receptor	NT-3 growth factor receptor precursor	Ntrk3	--	Q6VNS1	12569366	184
cell receptor	Tyrosine-protein kinase RYK precursor	Ryk	--	Q01887	19000841	231
cell receptor	Tyrosine-protein phosphatase non-receptor type substrate 1 precursor	Sirpa	--	P97797	24036914	253
cell receptor (CNTFR)	Ciliary neurotrophic factor receptor subunit alpha precursor	CNTFR	P26992	--	7681218	117
cell receptor (Eph)	Ephrin type-B receptor 1 precursor	EPHB1	P54762	--	12591933	315
cell receptor (Eph)	Ephrin type-B receptor 2 precursor	Ephb2	--	P54763	18713744	316
cell receptor (Eph)	Ephrin type-A receptor 7 precursor	Epha7	--	Q61772	22036564	314
cell receptor (Eph)	Ephrin type-A receptor 4 precursor	Epha4	--	Q03137	25264256	46
cell receptor (GPCR)	G-protein coupled receptor 126 precursor	GPR126	Q86SQ4	--	15189448	333
cell receptor (VPS10 domain)	VPS10 domain-containing receptor SorCS3 precursor	SORCS3	Q9UPU3	--	16393139	426
cell receptor (VPS10 domain)	Sortilin precursor	SORT1	Q99523	--	16393139, 21730062	426,204
cell receptor (VPS10 domain)	VPS10 domain-containing receptor SorCS2 precursor	SORCS2	Q96PQ0	--	16393139	426
cell receptor (VPS10 domain)	VPS10 domain-containing receptor SorCS1 precursor	SORCS1	Q8WY21	--	16393139	426
cell receptors	Plexin-B1 precursor	PLXNB1	O43157	--	12533544	188
cell receptors	Plexin-B2 precursor	PLXNB2	O15031	--	12533544	188
cell surface protein	Dyslexia-associated protein KIAA0319 precursor	KIAA0319	Q5VV43	--	20943657	123
cell surface protein	Myelin basic protein	MBP	P02686	P02688	2600084	379
growth factor binding protein	Cysteine-rich motor neuron 1 protein precursor	CRIM1	Q9NZV1	--	12805376, 25453556	298,299
guidance (axon)	Semaphorin-4B precursor	Sema4b	--	Q62179	23430253	72
guidance cue	Uncharacterized protein	SEMA5B	--	F1NSD7	22817385	95
Integral membrane protein	Integral membrane protein 2B	ITM2B	Q9Y287	--	21170088	234
Integral membrane protein	Amyloid-like protein 2 precursor	APLP2	Q06481	--	16279945, 12228233	266,265
Integral membrane protein	Amyloid-like protein 1 precursor	APLP1	P51693	--	9428684, 12228233	264,265
ligand	Ephrin type-A receptor 2 precursor	EPHA2	P29317	P52801	10958785	124
ligand	Ephrin-A5 precursor	Efna5	--	O08543	18951988, 16239146	23,125
ligand	Ephrin-B1 precursor	EFNB1	P98172	--	17567680	317
ligand	Gliomedin precursor	Gldn	Q8BMF8	--	17485493	326

	ligand	Delta-like protein 1 precursor	Dll1	--	Q61483	18342566, 18951988	301,23
	ligand	Leucine-rich repeat-containing protein 4B precursor	Lrrc4b	--	P0C192	24298159	157
	membrane transporters	Sodium- and chloride-dependent glycine transporter 2	Slc6a5	--	P58295	14675166	423
	neurotransmitter	Acetylcholinesterase precursor	ACHE	P22303	--	21214569	97
		Seizure 6-like protein 2 precursor	Sez6l2	--	Q4V9Z5	23430253	72
angiogenesis	cell adhesion molecule	Junctional adhesion molecule C precursor	JAM3	Q9BX67	--	20592283	51
	cell receptor	Tyrosine-protein kinase receptor Tie-1 precursor	TIE1	P35590	--	17728252	252
	cell receptor	Angiopoietin-1 receptor precursor	TEK	Q02763	--	17901375	143
	cell receptor	Vascular endothelial growth factor receptor 1 precursor	FLT1	P17948	--	19276374	232
	cell receptor	Urokinase plasminogen activator surface receptor precursor	PLAUR	Q03405	P35456	11342439	449
	cell receptor	Vascular endothelial growth factor receptor 2 precursor	KDR	P35968	--	20814017	233
	cell receptor	Endoglin precursor	ENG	P17813	--	20424116, 22296769	243,244
	cell receptor	Neuropilin-1 precursor	Nrp1	--	P97333	18818406	182
	cell receptor (GPCR)	G-protein coupled receptor 124 precursor	GPR124	Q96PE1	--	16982628	133
	cell receptor (GPCR)	Brain-specific angiogenesis inhibitor 1 precursor	BAI1	O14514	--	22330140	104
	growth factor receptor (EGFR)	C-type lectin domain family 14 member A precursor	CLEC14A	Q86T13	--	26939791	52
blood and homoeostasis	angiotensin	Renin receptor precursor	Atp6ap2	--	Q9CYN9	19380613	200
	iron homeostasis	Hemojuvelin precursor	HFE2	Q6ZVN8	--	20937842	134
	iron homeostasis	Transferrin receptor protein 1	TFRC	P02786	--	2752136	220
	iron homeostasis	Transferrin receptor protein 2	TFR2	Q9UP52	--	25637053	89
	platelet (cell surface protein)	Platelet glycoprotein V precursor	GP5	P40197	O08742	18951988	23
	platelet (cell surface protein)	Platelet glycoprotein VI precursor	GP6	Q9HCN6	--	24674813, 17445093	398,399
	platelet (cell surface protein)	Semaphorin-7A precursor	SEMA7A	O75326	--	20962327	73
	platelet (thrombus formation)	Semaphorin-4D precursor	SEMA4D	Q92854	--	17244710	202
	thrombin, coagulation	Thrombomodulin precursor	THBD	P07204	--	20605193	251
	vasoconstriction(cardiovascular system)	Type-1A angiotensin II receptor	Agtr1	--	P25095	20080964	94
cell growth and development	cell adhesion molecule (epithelial cell proliferation)	Epithelial cell adhesion molecule precursor	EPCAM	P16422	--	19136966, 24665393	59,60
	cell adhesion molecule (placental)	Protocadherin-12 precursor	PCDH12	Q9NPG4	--	21402705	195
	cell receptor (GPCR)	Beta-1 adrenergic receptor	ADRB1	P08588	--	23066091	76
	cell receptor (GPCR)	Endothelin B receptor precursor	EDNRB	P24530	--	12226103	313

cell receptor (GPCR)	Vasopressin V2 receptor	AVPR2	--	P48044	7896781	96
cell receptor (GPCR)	G-protein coupled receptor 56 precursor	GPR56	Q9Y653	--	25437104	334
cell receptor (GPCR)	Parathyroid hormone/parathyroid hormone-related peptide receptor precursor	PTH1R	Q03431	--	20080964	94
cell receptor (GPCR)	Proteinase-activated receptor 2 precursor	F2RL1	P55085	--	12594060	410
cell receptor (GPCR)	Proteinase-activated receptor 1 precursor	F2R	P25116	--	14982936	409
cell receptor (IGFR)	Insulin-like growth factor 1 receptor precursor	IGF1R	P08069	--	17524361	248
cell receptor (IGFR)	Cation-independent mannose-6-phosphate receptor precursor	IGF2R	P11717	--	2458716	100
cell receptor (NPR)	Atrial natriuretic peptide receptor 1 precursor	NPR1	P16066	--	2458716	100
cell receptor (SCFR)	Mast/stem cell growth factor receptor Kit precursor	KIT	P10721	--	14625290, 7536489	171,172
growth factor (EGFR)	Pro-epidermal growth factor precursor	EGF	P01133	P01132	14993236, 18951988	54,23
growth factor (EGFR)	Epigen precursor	Epgn	--	Q924X1	18951988, 17169360	23,126
growth factor (EGFR)	Probetacellulin precursor	BTC	P35070	Q05928	14993236, 11823465, 18951988	54,55,23
growth factor (EGFR)	Proepiregulin precursor	EREG	O14944	Q61521	19049889, 18951988	193,23
growth factor (ERBBs)	Pro-neuregulin-2, membrane-bound isoform precursor	NRG2	O14511	--	26027736	189
growth factor (ERBBs)	Pro-neuregulin-1, membrane-bound isoform precursor	NRG1	Q02297	Q6DR99	18951988, 20215529	23,407
growth factor (FGFR)	Fibroblast growth factor receptor 1 precursor	FGFR1	P11362	--	8692946	322
growth factor (FGFR)	Fibroblast growth factor receptor 2 precursor	FGFR2	P21802	--	16230393	127
growth factor (HGF)	Kunitz-type protease inhibitor 1 precursor	SPINT1	O43278	--	10544273	352
growth factor (HGFR)	Hepatocyte growth factor precursor	HGF	P14210	--	8117273	336
growth factor (kit)	Kit ligand precursor	KITLG	P21583	P20826	17344430	351
growth factor (VEGF)	Vascular endothelial growth factor A precursor	VEGFA	P15692	--	24501421	450
growth factor binding protein	Vasorin precursor	VASN	Q6EMK4	--	21170088	234
growth factor receptor	Tumor-associated calcium signal transducer 2 precursor	Tacstd2	--	Q8BGV3	20551327	227
growth factor receptor (ERBBs)	Receptor tyrosine-protein kinase erbB-4 precursor	ERBB4	Q15303	--	12475204	3
growth factor receptor (FGFR)	Fibroblast growth factor receptor 3 precursor	Fgfr3	--	Q61851	21865593	128
growth factor receptor (FGFR)	Fibroblast growth factor receptor-like 1 precursor	FGFRL1	Q8N441	--	19920134	245
growth factor receptor (HGFR)	Hepatocyte growth factor receptor precursor	MET	P08581	--	17875701, 23746173	337,338
growth factor receptor (NGFR)	Tumor necrosis factor receptor superfamily member 16 precursor	NGFR	P08138	P07174	14638693, 17904861	56,57
growth factor receptor (NGFR)	High affinity nerve growth factor receptor precursor	NTRK1	P04629	--	12058067	137

	growth factor receptor (TGF-beta R)	TGF-beta receptor type-1 precursor	TGFBR1	P36897	--	19595713	212
	growth factor receptor regulator	CD109 antigen precursor	CD109	Q6YHK3	--	20035377	284
	growth hormone receptor	Growth hormone receptor precursor	GHR	P10912		11309389	335
	growth hormone receptor (TSHR)	Thyrotropin receptor precursor	TSHR	P16473	--	18074395	213
	integral membrane protein	Leucine-rich repeats and immunoglobulin-like domains protein 3 precursor	LRIG3	Q6UXM1	--	20041192	132
	integral membrane protein	Leucine-rich repeats and immunoglobulin-like domains protein 1 precursor	LRIG1	Q96JA1	--	21087604	158
	ion channel	Acid-sensing ion channel 1	ASIC1	P78348	--	20601429	75
	ion channel	Amiloride-sensitive sodium channel subunit gamma	Scnn1g	--	Q9WU39	15007080, 18650438	259,260
	ion channel	Sodium channel subunit beta-3 precursor	Scn3b	--	Q8BHK2	15824102, 18694383	424,425
	ion channel	Sodium channel subunit beta-4 precursor	Scn4b	--	Q7M729	15824102, 18694383	424,425
	ion channel	Amiloride-sensitive sodium channel subunit alpha	Scnn1a	--	Q61180	15007080	259
	ion channel	Sodium channel subunit beta-2 precursor	Scn2b	--	Q56A07	15824102, 18694383	424,425
	ion channel	Sodium channel subunit beta-1 precursor	Scn1b	--	P97952	15824102, 18694383	424,425
	ion channel	Calcium-activated chloride channel regulator 4A precursor	Clca4a	--	Q6Q473	22350745	30
	ligand	Ectodysplasin-A	EDA	Q92838	--	11309369	58
	morphogens	Sonic hedgehog protein precursor	SHH	Q15465	--	23118222	61,62
lipid	adipocyte factor (adipogenesis)	Protein delta homolog 1 precursor	DLK1	P80370	Q09163	16809777, 26312857	164,165
	cell receptor (lipoprotein receptor famiy)	Low-density lipoprotein receptor-related protein 8 precursor	LRP8	Q14114	--	25233900, 15950758	165
	cell receptor (lipoprotein receptor famiy)	Very low-density lipoprotein receptor precursor	VLDLR	P98155	--	15950758	163
	cell receptor (lipoprotein receptor famiy)	Low-density lipoprotein receptor-related protein 1B precursor	LRP1B	Q9NZR2	Q9JI18	17227771	358
	cell receptor (lipoprotein receptor famiy)	Low-density lipoprotein receptor precursor	LDLR	P01130	--	3380796	360
	cell receptor (lipoprotein receptor famiy)	Low-density lipoprotein receptor-related protein 6 precursor	LRP6	O75581	--	17326769	163
	cell receptor (lipoprotein receptor famiy)	Low-density lipoprotein receptor-related protein 4 precursor	Lrp4	--	Q8VI56	17227771	359
	cell receptor (lipoprotein receptor famiy)	Low-density lipoprotein receptor-related protein 2 precursor	Lrp2	--	P98158	15180987	406
	cell receptor (metabolism)	Prolow-density lipoprotein receptor-related protein 1 precursor	LRP1	Q07954	--	19371428	63,368
melanogenesis	melanosome	Melanocyte protein PMEL precursor	PMEL	P40967	--	19047044, 19884326	64,65
insulin	cell surface protein	Collectrin precursor	TMEM27	Q9HBJ8	--	22628310, 16330324	154
	cell surface protein	Kin of IRRE-like protein 2 precursor	Kirrel2	--	Q7TSU7	26324709	281
renal	cell receptor	Polycystin-1 precursor	PKD1	P98161	--	17525154	401

	urine	Uromodulin precursor	UMOD	P07911	--	11741296	66
enzyme	aminopeptidase	Leucyl-cystinyl aminopeptidase	LNPEP	Q9UIQ6	--	14751233	353
	carboxypeptidase	Angiotensin-converting enzyme precursor	ACE	P12821	--	10187843	67
	decarboxylase	Glutamate decarboxylase 2	Gad2	--	Q05683	16879709	327
	enzyme Inducer (MMP Inducer)	Basigin precursor	BSG	P35613	P26453	14985463, 17050542, 1714914	273,274,275
	enzyme Inhinitor (peptidase inhibitor)	Reversion-inducing cysteine-rich protein with Kazal motifs precursor	RECK	O95980	--	19022775	421
	nucleotidase	5'-nucleotidase	NT5E	P21589	--	9015312	256
	peptidase	Endothelin-converting enzyme 1	ECE1	P42892	--	17761169	82
	peptidase	Dipeptidase 1 precursor	DPEP1	P16444	--	11988094	303
	peptidase	Neprilysin	MME	P08473	--	24495806	385
	protease	72 kDa type IV collagenase precursor	MMP2	P08253	--	8663332	257
	protease	Matrix metalloproteinase-14 precursor	MMP14	P50281	--	15137052	365
	protease (beta-secretase)	Beta-secretase 1 precursor	BACE1	P56817	P56818	12857759	276
	protease (beta-secretase)	Beta-secretase 2 precursor	BACE2	Q9Y5Z0	--	11316808	277
	protease (metalloproteinase)	Disintegrin and metalloproteinase domain-containing protein 10 precursor	ADAM10	O14672	O35598	26554003	307
	protease (metalloproteinase)	Disintegrin and metalloproteinase domain-containing protein 15 precursor	ADAM15	Q13444	--	25208722	304
	protease (metalloproteinase)	Disintegrin and metalloproteinase domain-containing protein 19 precursor	ADAM19	Q9H013	--	12393862	305
	protease (metalloproteinase)	Disintegrin and metalloproteinase domain-containing protein 28 precursor	ADAM28	Q9UKQ2	O35598	15013428, 26554003, 23643150	306,307,308
	protease (metalloproteinase)	Disintegrin and metalloproteinase domain-containing protein 8 precursor	ADAM8	P78325	Q05910	12372841	309,310
	protease (metalloproteinase)	Meprin A subunit alpha precursor	MEP1A	Q16819	P28825	25617491, 9439598	371,238
	protease (metalloproteinase)	Meprin A subunit beta precursor	MEP1B	Q16820	--	25617491, 12941954	371,372
	protease (serine peptidase)	Atrial natriuretic peptide-converting enzyme	CORIN	Q9Y5Q5	--	21288900	271
	protease (serine peptidase)	Suppressor of tumorigenicity 14 protein	ST14	Q9Y5Y6	--	11231297	428
	protease (metalloproteinase)	Stromelysin-1 precursor	MMP3	P08254	--	14681236	427
	proteinase	Furin precursor	FURIN	P09958	--	12220680, 11237874	324,325

	proteinase inhibitor	Tissue factor pathway inhibitor precursor	TFPI	P10646	--	10859319	436
	receptor tyrosine kinase	Tyrosine-protein kinase receptor UFO precursor	AXL	P30530	Q00993	7822279, 16227584	229,230
	receptor-like PTPases	Receptor-type tyrosine-protein phosphatase F precursor	PTPRF	P10586	--	16478662	197
	receptor-type PTPases	Receptor-type tyrosine-protein phosphatase mu precursor	PTPRM	P28827	--	8620001, 19690139	419,420
	receptor-type PTPases	Receptor-type tyrosine-protein phosphatase S precursor	Ptprs	--	Q64605	9245795	199
	receptor-type PTPases	Receptor-type tyrosine-protein phosphatase R precursor	Ptprr	--	Q62132	17147696	198
	receptor-type PTPases	Receptor-type tyrosine-protein phosphatase alpha precursor	Ptpra	--	P18052	18713734	196
	receptor-type PTPases	Receptor-type tyrosine-protein phosphatase zeta precursor	Ptprz1	--	B9EKR1	18713734	196
	ribosyltransferase	T-cell ecto-ADP-ribosyltransferase 1 precursor	Art2a	--	P17981	26209623	205
	transferase	Beta-galactoside alpha-2,6-sialyltransferase 1	St6gal1	--	P13721	2505760	77
	transferase	Beta-1,4-galactosyltransferase 1	B4GALT1	P15291	--	3093147, 17021253	102,103
transporter	folate transporter	Folate receptor alpha precursor	FOLR1	P15328	--	1846624	68
	ion transporter	Zinc transporter ZIP10 precursor	Slc39a10	--	Q6P5F6	22687393	236
	ion transporter	Zinc transporter ZIP4 precursor	Slc39a4	--	Q78IQ7	18936158	237
	protein transporter	Golgi integral membrane protein 4	GOLIM4	O00461	--	20041192	132
cell surface structure	--	Collagen alpha-1(XIII) chain	COL13A1	Q5TAT6	--	15005656	291
	--	Collagen alpha-1(XXIII) chain	COL23A1	Q86Y22	Q8K4G2	17627939, 12644459	80,81
	collagen receptor	Epithelial discoidin domain-containing receptor 1 precursor	DDR1	Q08345	--	16440311	320
	desmosome (cell junction)	Desmocollin-3 precursor	DSC3	Q14574	--	11500511, 24665393	302,60
	desmosome (cell junction)	Desmoglein-3 precursor	DSG3	P32926	--	11500511, 24665393	302,60
	desmosome (cell junction)	Desmoglein-2 precursor	DSG2	Q14126	--	26224314	69
	desmosome (cell junction)	Desmoglein-1 precursor	DSG1	Q02413	--	26224314, 24665393	69,60
	dystroglycan complex	Dystroglycan precursor	DAG1	Q14118	Q62165	19946898, 16701552	311
	hemidesmosome (cell junction)	Collagen alpha-1(XVII) chain	COL17A1	Q9UMD9	--	17545155	292
	mucous barriers (cell surface protein)	Mucin-1 precursor	MUC1	P15941	--	12441351, 15130087	377,378
	outer segments (os)	Cadherin-related family member 1 precursor	Cdhr1	--	Q8VHP6	15284225	105
	proteoglycan	Glypican-1 precursor	GPC1	--	P50593	20008810	330
	proteoglycan	Syndecan-3	SDC3	O75056	P33671	12929127, 14504279	430,431
	proteoglycan	Chondroitin sulfate proteoglycan 4 precursor	CSPG4	Q6UVK1	Q00657	15866049	290
	proteoglycan	C-type lectin domain family 3 member A precursor	CLEC3A	O75596	--	19173304	297
	proteoglycan	Chondroitin sulfate proteoglycan 5 precursor	Cspg5	--	Q9ERQ6	17532789	242

	tight junction (cell junction)	Claudin-2	CLDN2	P57739	--	16232214, 24665393	78,60
	tight junction (cell junction)	Claudin-5	Cldn5	--	O54942	26242473	79
	tight junction (cell junction)	Occludin	OCLN	Q16625	--	17038551,15472219, 24665393	393,394,60
ageing	anti-aging	Klotho precursor	KL	Q9UEF7	--	25110992,19737556	70,71
